# Breastfeeding among women employed in Mexico’s informal sector: strategies to overcome key barriers

**DOI:** 10.1186/s12939-024-02147-x

**Published:** 2024-07-23

**Authors:** Julia M. Goodman, Vania Lara-Mejía, Sonia Hernández-Cordero, Mireya Vilar-Compte

**Affiliations:** 1 OHSU-PSU School of Public Health, Vanport Building, Ste. 510, 97201 Portland, OR USA; 2 Instituto de Investigaciones para el Desarrollo con Equidad (EQUIDE), Universidad Iberoamericana, Ciudad de México, Mexico; 3 Department of Public Health, Montclair State University, Montclair, NJ USA

**Keywords:** Breastfeeding, Maternity leave, Informal employment, Public policy, Mexico

## Abstract

**Background:**

Rates of exclusive breastfeeding fall below recommended levels, particularly among women in paid employment. In Mexico, more than half of women are in informal employment, meaning they lack many of the protections that may support breastfeeding.

**Methods:**

In-depth interviews with 15 key informants representing government agencies (*n* = 6 organizations), NGOs (*n* = 4), international organizations (*n* = 2), and academia (*n* = 2) in Mexico. Interviews were conducted between March and June 2023. To understand and describe barriers to breastfeeding among informally employed women in Mexico according to key informants and the current and potential policies to address these barriers, we conducted a qualitative thematic analysis.

**Results:**

Current policies to promote, protect, and support breastfeeding predominantly apply to all employed women, but respondents expressed concern that they did not provide adequate protection for women in informal employment. Additional themes concerned the need for relevant programs to be institutionalized and coordinated, discussions of breastfeeding as a right, and the legal equivalence (whether true in practice or not) of formal and informal workers.

**Conclusions:**

Women employed in Mexico’s informal sector face a dearth of maternity protections. According to key informants, few policies exist to promote, protect, and support breastfeeding among employed women, in general, but the economic vulnerability and challenging working conditions of women in informal employment exacerbates their situation. The lack of access to formal labor protections, such as paid maternity leave, creates a significant barrier to breastfeeding for women in the informal sector. Recommendations include short-term policies to fill gaps in social protection for informally employed women, as well as longer-term solutions such as the development of universal social protection programs and supporting formalization.

**Supplementary Information:**

The online version contains supplementary material available at 10.1186/s12939-024-02147-x.

## Background

The health benefits of breastfeeding are well documented and include healthy brain development, reduced risk of obesity and chronic health conditions, and birth spacing [1]. As a result, the World Health Organization (WHO) and UNICEF recommend exclusive breastfeeding until infants are at least 6 months old and continuation of breastfeeding until at least 2 years of age once complementary foods are introduced [2, 3]. 

In Mexico, breastfeeding indicators fall below recommended levels, particularly among women in paid employment [4–6]. According to the 2021–2022 National Health and Nutrition Survey (ENSANUT, for its Spanish name), just 33.6% of infants received exclusive breastmilk until at least 6 months of age– significantly lower than both the global average of 48% and WHO’s goal of 70% by 2030 [6, 7]. Differences in breastfeeding indicators have been found between women in paid and unpaid employment. Mexican women with a paid employment (formal or informal) were 20% less likely to ever breastfeed (*p* < 0.01), 10% less likely to have an early initiation of breastfeeding (*p* < 0.001) and 20% less likely to exclusively breastfeed (*p* < 0.05) compared with women without a paid employment [4]. Much evidence suggests that return to work is one of the main reasons for breastfeeding cessation [8–10]. As such, strategies to promote and support breastfeeding include paid maternity leave, reduced work hours, and break time for lactation [11–13]. Workplace policies and strategies to promote and support breastfeeding not only contribute to the nutrition and health of children and mothers but are also beneficial to companies. On the one hand, they can reduce absenteeism and healthcare costs; on the other, they can increase employee retention, productivity, and loyalty [14]. 

Among employed women, those in informal employment are often the most economically vulnerable [15]. The International Labour Organization defines informal employment as that which, in law or in practice, is not covered or is insufficiently covered by formal arrangements [16]. In Mexico, this most often refers to jobs that are not connected with a national social security program, for example, the Mexican Social Security Institute (IMSS) or the Institute for Social Security and Services for State Workers (ISSSTE). In 2023, it was estimated that 56% of women in Mexico were in informal employment [17]. While imperfect, IMSS and ISSSTE provide a basic safety net for formal sector workers, including healthcare, childcare, and a public pension. Further, they provide a mechanism for paid maternity leave.

According to the Mexican Constitution and Federal Labor Law, employed women in Mexico who have given birth are entitled to 12 weeks of paid maternity leave, guaranteeing their full salary and preserving their employment and legal benefits. Following the end of maternity leave, they are entitled to two extra breaks of 30 min each in order to breastfeed or express breastmilk at a designated place, or a reduction of one hour in the workday [18, 19]. Informally employed women are not explicitly excluded from these social protections but, by definition, they are not enrolled in the systems necessary for distributing benefits (e.g., IMSS, ISSSTE) [20]. Therefore, they are effectively left without access to any paid maternity leave and childcare. The lack of social protection is an organizational and community barrier that interferes with women’s ability and right to optimally breastfeed [21]. Another important community barrier is cultural beliefs. One study showed that living in an environment where breastfeeding is stigmatized in public places influenced Mexican women’s decisions about their infant feeding practices [22]. 

Given the documented importance of maternity leave for supporting breastfeeding, extending maternity leave to women in the informal sector emerged as a key recommendation from a Mexican breastfeeding expert committee in 2017 [23]. One mechanism for achieving this could be through a noncontributory maternity cash transfer program, as this intervention aims to address women’s income, a key social determinant of health [24]. Recent costing estimates from Mexico suggest that a maternity cash transfer program would be comparable to similar social protection policies already in place [21]. Cash transfer programs are sound nutrition-sensitive interventions that can help to achieve the nutrition outcomes linked to the Sustainable Development Goals (SDG), more specifically SDG 2.2 for which one key indicator is exclusive breastfeeding. In Latin America cash transfers have been widely implemented to improve the material conditions of people living in poverty, often with behavioral conditions attached that incentivize investment in children’s health and wellbeing. For example, in Mexico, between 1997 and 2019 a conditional cash transfer program provided income support to households living in poverty while requiring parents to enroll their children in school and attend periodic health check-ups [25]. However, such social protection mechanisms have seldom been used to explicitly promote and protect breastfeeding by financially supporting mothers in ways resembling maternity leave.

Mexico’s effort to improve infant feeding practices was initiated after the sharp decrease in breastfeeding practices, as shown by the National Health and Nutrition Survey 2012. As a result, key actors, including organizations in civil society, academia, and government started to identify strategies and opportunities to improve breastfeeding in the country [26]. Despite efforts to improve breastfeeding practices in Mexico, there are many areas for improvement of the actions established for this purpose in the country [23]. 

Increasing access to programs and policies to protect, promote, and support breastfeeding among informally employed women has been suggested as a mechanism for social justice [21]; however, little research has examined which policies and programs have the most potential to be implemented. As a first step, this paper focuses on the perceptions of key informants who are engaged in advocacy, design and/or implementation of programs and policies related to breastfeeding or informal employment in Mexico. The purpose of this study is to describe how key informants understand the barriers to breastfeeding among women employed in Mexico’s informal sector and to document existing and potential labor policies and programs that promote, protect, and support breastfeeding among these women.

## Methods

### Study design and participants

This study was conducted in Mexico City, Mexico in March-June 2023. The study employed a descriptive qualitative study design. Participants were key informants from governmental agencies, civil society, and academic institutions that were identified as engaging in work relevant to breastfeeding or informal employment in Mexico. An initial list of potential participants was developed by the members of the study team (SHC, MVC, and VLM) with extensive knowledge of breastfeeding policies in Mexico and was informed by a recent NetMap analysis of actors involved in the design and implementation of workplace breastfeeding interventions in Mexico (e.g., IMSS: Mexican Institute of Social Security; STPS: Ministry of Labor and Social Welfare; and UNICEF) [27]. We then supplemented this list with organizations that focus on informal employment, particularly those with an emphasis on supporting women (e.g., WIEGO: Women in Informal Employment Globalizing and Organizing). We identified individuals within organizations based on their knowledge, experience, and/or position within the organization. Snowball sampling was then employed to identify additional participants. Informal invitations, including a short description of the study, were sent via e-mail by the co-authors (JMG, VLM, SHC) to solicit general interest for participation. Subsequently, invitees that expressed interest in interview participation received a formal invitation and an informed consent form by email. A total of 15 representatives from government agencies (*n* = 6 organizations), NGOs (*n* = 4), international organizations (*n* = 2), and academia (*n* = 2) participated in the study (Table 1). We contacted an additional 2 governmental agencies, 3 NGOs, and 1 international organization but individuals either did not respond (*n* = 5) or declined to be interviewed (*n* = 1). Summaries of each participating organization and how their work relates to breastfeeding and/or informal employment are in Additional file 1.

**Table 1:** Participant characteristics by sector

Actor group	Number of interviews	Organizations represented	
Government	6	INMujeres, IMSS, STPS, CNEGSR*, Secretaría de Bienestar, SNDIF	
Civil Society organizations	4	Pacto por la Primera Infancia, ACCLAM, Infancia Plena, CEEY	
International organizations	2	WIEGO, UNICEF	
Academia	2	IBERO, INSP	
Total	14		

ACCLAM: Association of International Board Certified Lactation Consultants in Mexico; CEEY: Espinosa Yglesias Study Center; CNEGSR: National Center for Gender Equity and Reproductive Health; IBERO: Universidad Iberoamericana Mexico City; IMSS: Mexican Institute of Social Security; INMujeres: National Women’s Institute; INSP: National Public Health Institute; SNDIF: National System for the Integral Development of the Family; STPS: Ministry of Labor and Social Welfare, UNICEF: United Nations Children’s Fund; WIEGO: Women in Informal Employment Globalizing and Organizing.

* The CNEGSR interview had two participants

### Data collection

In-depth interviews were conducted through the Zoom platform using a semi-structured interview guide that was developed based on the research questions (see Additional file 2 for the interview guide). Interviews asked respondents to describe the population of women and the jobs they perform in Mexico’s informal sector, barriers to breastfeeding these women face, and current and potential policies to promote, protect, and support breastfeeding. We also asked respondents about their perspectives on a maternity cash transfer program, as this has been proposed in the literature, including recent studies to estimate the cost of implementing such a program in Mexico [21, 28]. We present these results as a “case study” to differentiate them from current and potential policies that respondents identified organically. Upon request, the interviewees were informed of the guiding interview questions prior to the interview. In cases where something was unclear or we wanted additional information, we emailed follow up questions to interviewees after the initial interview. All but one of the interviews were conducted with one interviewee. One interview was conducted with two interview partners following the request of the participating organization. Interviews lasted approximately 50 to 60 min each. All participants agreed to the interviews being audio recorded. After each interview, the audio recordings were transcribed verbatim using Sonix.ai software and checked for accuracy by one researcher (VLM).

### Data analysis

To understand and describe participants’ perceptions of barriers to breastfeeding among informally employed women in Mexico and the current and potential policies to address these barriers, we conducted a qualitative thematic analysis. We used Dedoose (version 9.0.78) to manage and analyze the data. Two researchers (JMG and VLM) developed the codebook. The codebook included a combination of *a priori* codes that were developed deductively based on the research questions (e.g., characteristics of women employed in Mexico’s informal sector, barriers to breastfeeding in this population, current policies/programs, potential policies/programs, barriers to formalization) and the Socio-Ecological Model (SEM) [29, 30] used to guide the analysis and inductive codes that emerged during the interviews (e.g., legal equivalence between formal and informal employment, right to breastfeed). These two researchers jointly coded one interview and discussed code application and additions to the codebook. The final codebook with examples of each code is in Additional file 3. For the remaining interviews, one researcher coded each interview (VLM) and a second researcher (JMG) reviewed the coded transcriptions and added/edited codes. Differences in code application were resolved through discussion. The researchers took detailed notes and compiled memos throughout the research process. All analysis was conducted with the original Spanish-language transcriptions. Selected quotes were then translated into English for presentation in this manuscript (see Additional file 4 for original Spanish-language quotes).

### Ethical approval and consent to participate

The Institutional Review Boards of Iberoamericana University (Mexico City, Mexico) and Portland State University (Oregon, USA) approved this study. A signed written informed consent was obtained from all interview participants. To guarantee the confidentiality and anonymity of the information, each interviewee was identified with an ID that replaced her or his name in the interview transcript.

## Results^1^

### Respondent characteristics

Fifteen respondents representing 14 organizations participated in our study (Table 1). Respondents were 80% female with an average age of 42.9 and an average job tenure of 4.4 years (Table 2). Most lived in Mexico City with two in neighboring states (Morelos, Mexico State) and one in the northern border state of Nuevo León. Respondents held a range of relatively senior positions within their organizations, including coordinators, directors, and area heads; however, most were managerial positions without the authority to make policy decisions.

**Table 2:** Participant characteristics by individual

Characteristic	Total (*n* = 15)*
Sex (%)	
Female	12 (80.0)
Male	3 (20.0)
Average age (Years) (SD)	42.9 (± 10.8)
Average time in current position (Years) (SD)	4.4 (± 4.7)
State of residence (%)	
Mexico City	12 (80.0)
Mexico State	1 (6.7)
Morelos	1 (6.7)
Nuevo León	

* The CNEGSR interview had two participants

### Characteristics of women employed in Mexico’s informal sector and their jobs, according to key informants

**Fig. 1:**
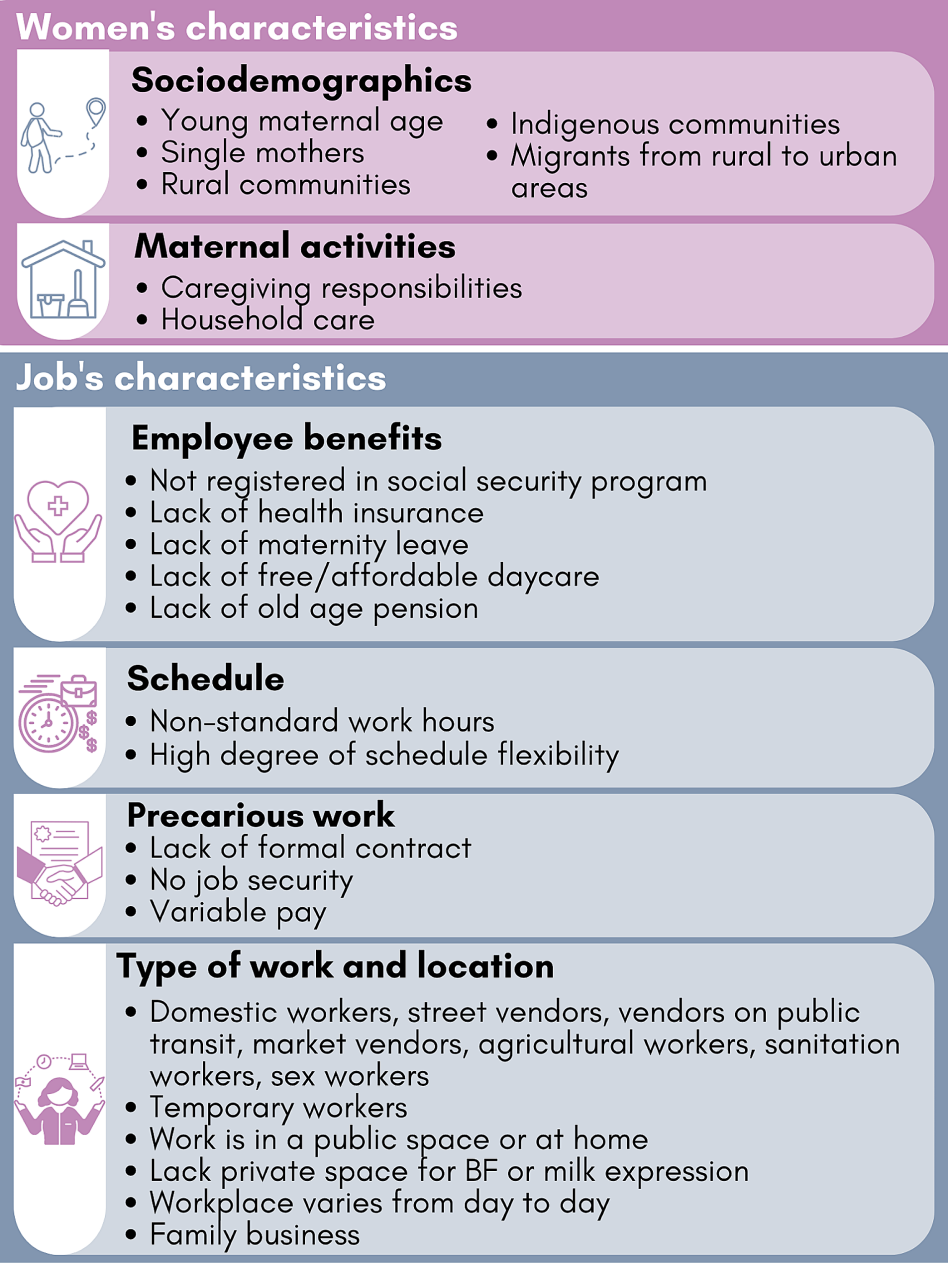
Characteristics of informally employed women and their jobs, according to key informants

When asked to describe who women employed in Mexico’s informal sector are, respondents described both characteristics of women themselves and characteristics of the informal jobs they may hold (Fig. 1).

#### Women’s characteristics

Overwhelmingly, respondents mentioned unpaid caregiving and domestic work as being key factors in women seeking or maintaining informal work. Several respondents described two distinct “types” of women with informal work in Mexico: women with high socioeconomic status (SES) who seek flexibility in order to care for young children and who face insufficient support in formal sector jobs; and women with lower SES who also sought flexibility but who had few options in the formal sector. Discussions mostly focused on the latter type of worker, who were also described as more likely to be young, single mothers, living in rural communities or having migrated from rural to urban areas, and disproportionately from Indigenous communities in Mexico.

#### Informal Job characteristics

Workers who were not eligible to register with a national social security program (e.g., IMSS, ISSSTE) that provides health insurance, 12 weeks of paid maternity leave, free childcare, an old age pension, and other related benefits, were characterized as informally employed. However, several respondents emphasized that informal work could also extend to precarious work, or work that did not have a formal contract or job security. Respondents also emphasized that individuals could be employed informally (e.g., without a contract or employee benefits) within formal organizations.

*“…In Mexico, I believe that informality masks a very ****high level of precarity****among workers. In reality, it is precarious work, more than informal work […] It would seem that when we say informal […] the only thing missing is a contract. When in fact what is happening in informality is a very high level of precarity. And then this precarity produces a great deal of inequality in relation to other workers, who are formal, but it is also very much affected by the gender order. In Mexico, there is a very low incorporation of women into the formal labor market…” (Female, Government, [01G])*

Respondents described occupations that represent a large proportion of informal jobs: domestic work, street vendors, market vendors, vendors on transit, agricultural workers, sanitation workers, and sex workers. Referring to those higher SES women in informal employment, respondents also discussed entrepreneurs as an important category of informal workers. Informal jobs were often described as taking place in public spaces (e.g., on public transit, on city streets) and/or in locations that may vary from day to day, which made it unlikely these women would have a private place to breastfeed or express breast milk. However, other informal jobs were described as taking place within one’s home or a family-owned business, providing flexibility and privacy for breastfeeding.

### Barriers to breastfeeding among women employed in Mexico’s informal sector, according to key informants

**Fig. 2:**
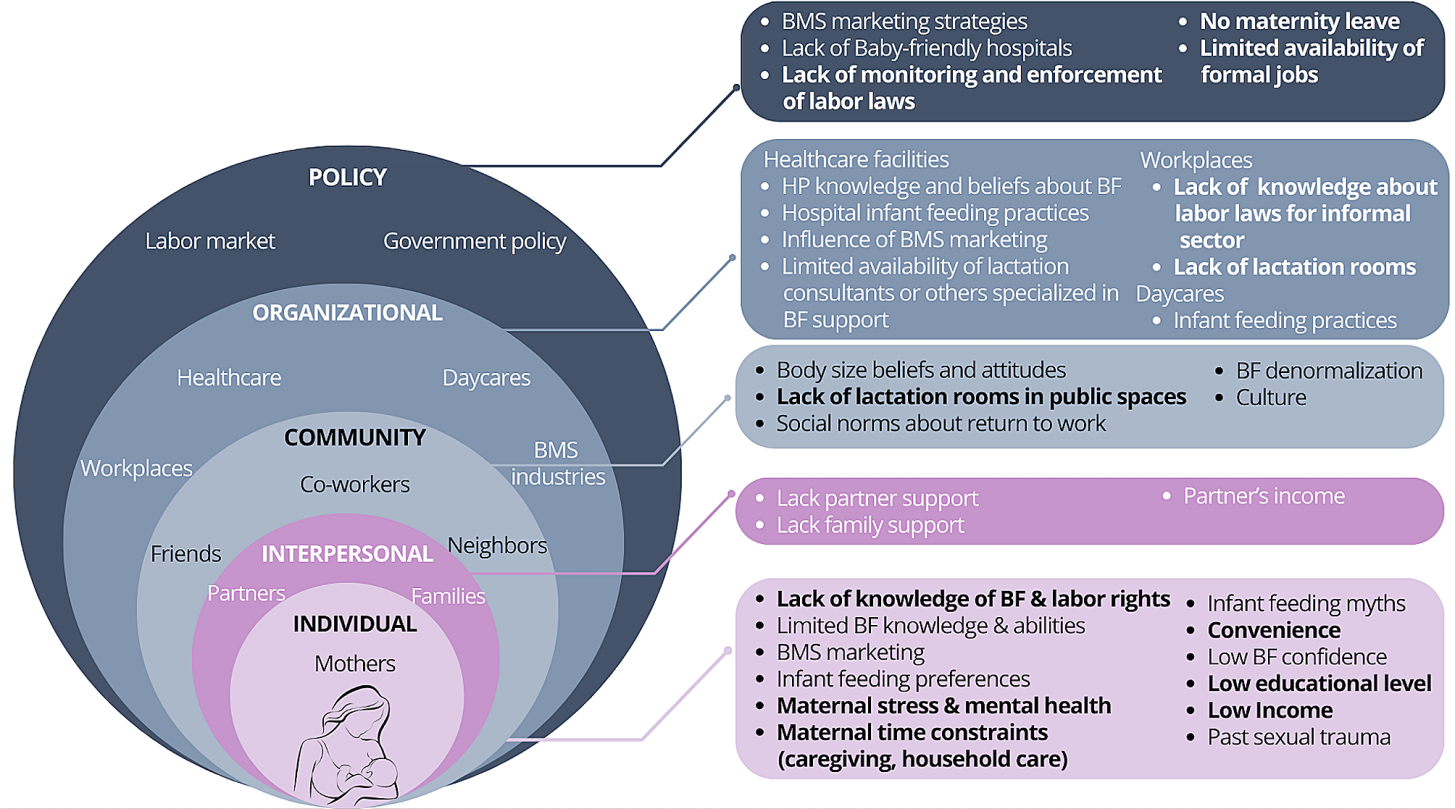
Barriers to breastfeeding among informally employed women according to key informants based on the Socio-Ecological Model (SEM), according to key informants. *Note* Bold indicates barriers that are more pronounced among women in informal employment. BMS: breast-milk substitutes; HP: health professionals; BF: breastfeeding.

While many barriers to breastfeeding could apply to all workers, such as commercial milk formula marketing and the lack of partner support for breastfeeding, others were either more pronounced among or exclusive to workers in the informal sector. Respondents mentioned that some barriers, such as misinformation, span different levels of influence, which can be identified in the SEM (Fig. 2).

*“…I believe that ****misinformation is a cross-cutting barrier***, *because when legislators do not have information, when budget makers do not have information, those who make public policies have no information, whoever you meet in the subway line does not have information, your right to breastfeeding is going to be wholesale trampled…” (Female, Civil Society Organization, [03 C])*

Given the increased barriers to breastfeeding at all levels of the SEM among informally employed women, the lack of formal jobs available was described as a significant barrier to breastfeeding at the policy level. Another key policy-level barrier was the lack of paid maternity leave that resulted from informally employed women being left out of IMSS.

While paid leave is not available to workers who are not registered with federal social security programs (e.g., IMSS), the Mexican Constitution and Federal Labor Law guarantee paid maternity leave and break time for breastfeeding to all workers, regardless of sector. However, respondents identified the lack of monitoring and enforcement of labor laws as a key policy-level barrier. A related organizational level barrier was that many employers were described as being unaware that federal labor laws applied to informally employed workers. Moreover, many respondents themselves were unaware that these laws applied to informal workers.

*“…And the other thing, which also seems to me to be nonsense on the part of the government, is to say that in informality there are no rights. This is legally false because if one reviews the legal framework of human rights when it talks about the ****right to work****[…] It doesn’t say formality or informality […] In other words, people who work have these rights. So, what corresponds to the State is to extend the rights of working people to the informal sector as well…” (Female, Civil society organizations, [06 C])*

The lack of lactation rooms in workplaces and public spaces was described as a key barrier at the organizational and community levels, respectively.

At the individual level, knowledge about rights related to breastfeeding and employment may be more limited among informal workers relative to workers in formal employment. Moreover, lower educational attainment and income among informal workers may contribute to increased stress and time constraints, all of which may pose challenges to breastfeeding.

*“…So, for me it places them in a state of vulnerability, especially because of the issue of economic resources. And that would trigger for me a lot of things like ****stress and then mental health problems***. *And a lot of things…” (Female, Civil society organizations, [01 C])*

Some respondents mentioned the challenge informal workers face in affording materials for the expression and storage of breast milk, for example.

*“…Another barrier is that, of course, the equipment for storing and transporting breastmilk also has a cost that women are ****not always able to pay***…*” (Male, International Organization, [04 C])*

### Barriers to formalization for women, according to key informants

Informality itself was a key barrier to breastfeeding across all levels of the SEM. Therefore, we asked respondents to discuss what they see as the primary factors that prevent formalization. Gendered expectations about caregiving and household work, resulting in “time poverty”, were described as key barriers preventing women from moving into formal employment.

*“…In Mexico, a woman can ****dedicate up to 40 hours a week to unpaid activities ****within the home. In domestic chores, caring for children, sick or elderly people. And this is almost equivalent to a full-time job…” (Male, Government, [06G])*

Specifically, the lack of flexibility in many formal sector jobs was described as a barrier for women with caregiving responsibilities.

*“…And if they are mothers responsible for their families, mothers or heads of household, they cannot take a formal job because it is from 9 to 5 if all goes well or from 9 to 7 and they have no help, right? So that pushes them to work in informal sectors that maybe they haven’t worked in all their lives […] but they were single mothers who need to take care of their children, who do not have any kind of help and who need ****certain schedules and flexibility that they do not have in the formal sector***…*” (Female, Government, [03G])*

The lack of educational opportunities was mentioned as another key barrier to formalization.

*“…Their ****educational level does not allow ****them to join formality…” (Female, Academia, [01 A])*

Respondents also highlighted the lack of high-quality jobs in the formal sector, particularly in rural communities. These respondents stated that even if every woman wanted to move into formal employment, there would not be sufficient opportunities.

*“…I bet you that if tomorrow all these people decided to wake up and look for a formal job, there would ****not be enough formal jobs for these people****[…] for all the people who work in informal employment…” (Female, Civil Society Organization, [06 C])*

### Policies/programs to promote, protect, and support breastfeeding

**Fig. 3:**
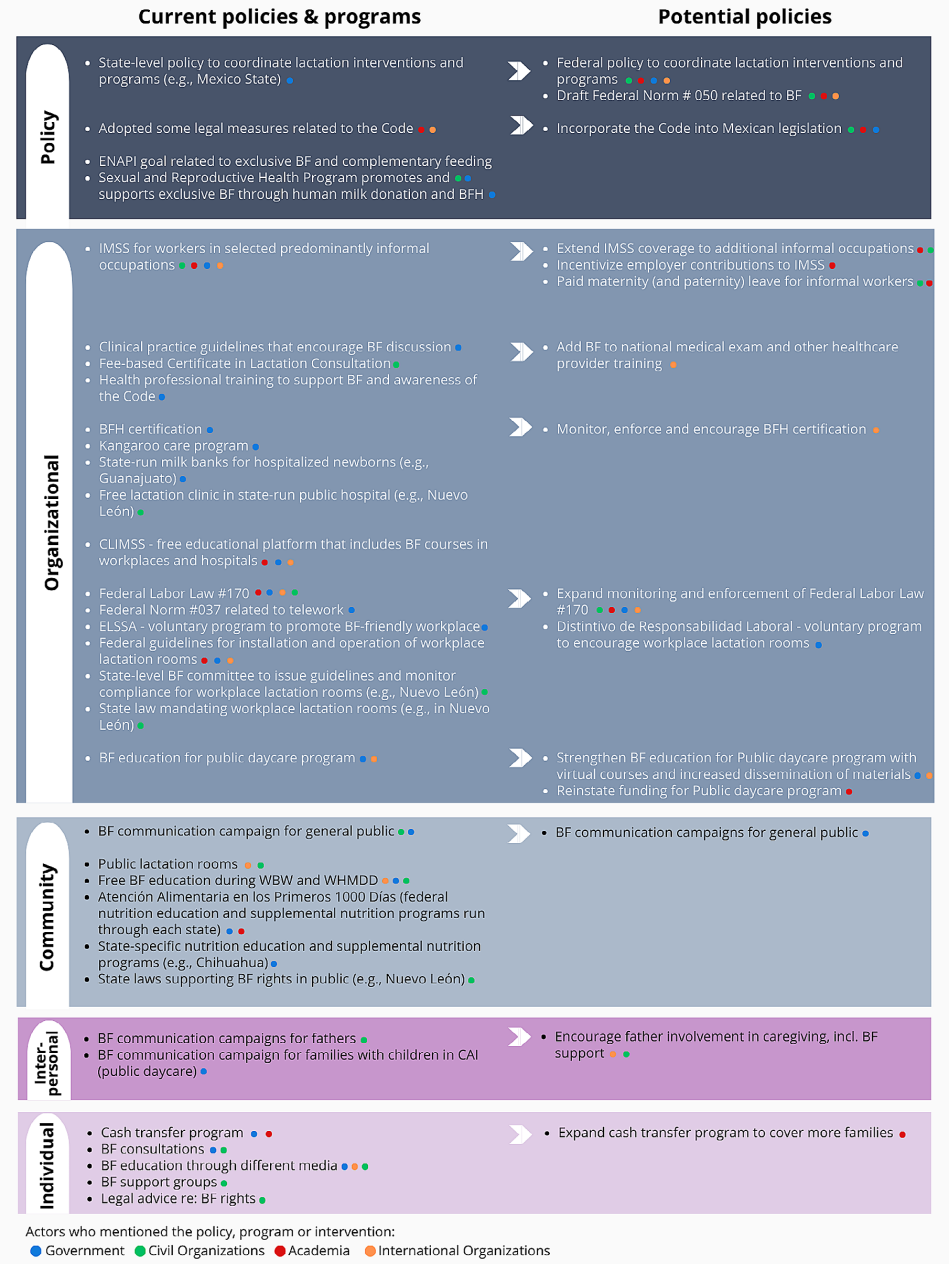
Current and potential policies to promote, protect, and support breastfeeding among informally employed women in Mexico based on the Socio-Ecological Model (SEM), as mentioned by key informants The Code: International Code of Marketing of Breast-Milk Substitutes; ENAPI: Estrategia Nacional de Atención a la Primera Infancia; BF: breastfeeding; BFH: Baby-Friendly Hospital; IMSS: Instituto Mexicano del Seguro Social; CLIMSS: Cursos en Línea del Instituto Mexicano del Seguro Social; ELSSA: Entornos Laborales Seguros y Saludables; WBW: World Breastfeeding Weak; WHMDD: World Human Milk Donation Day; CAI: Centro de Atención Infantil.

Figure 3 shows the current and potential policies and programs that respondents mentioned as promoting, protecting, and supporting breastfeeding, arranged according to the SEM. Six key themes emerged in discussions of these policies.

#### Theme 1: Potential policies as expansions of current policies/programs

Most potential policies were extensions of existing policies, indicated with an arrow in Fig. 3. At the policy level, which we categorized as policies or programs that focused on strategy, coordination, or adoption of the International Code of Marketing of Breast-Milk Substitutes (“the Code”), expansions included, for example, moving from adopting some legal measures related to the Code to incorporating the Code into Mexican legislation. Another example was a state-level policy to coordinate lactation interventions and programs, which then was discussed as a policy that could be implemented at the federal level to impact lactation across the country.

Several respondents discussed the ongoing effort to implement a National Care System as potentially having a major role in supporting breastfeeding among women in Mexico’s informal sector due to its potential impact on social co-responsibility for care. The National Care System that has been proposed builds on similar systems that have been promoted by feminist movements across Latin America (though does not have an immediate precursor in Mexico) and emphasizes the right to care as a human right. One respondent described Mexico’s proposed National Care System as follows:

*“…What we have in Mexico today is a set of actions of disjointed practices, oriented towards care, such as, for example, childcare centers, such as full-time schools for the care of school-aged children. Some other policies or measures that have been established in the law, such as palliative care units for people with terminal illnesses. […] This, well, all this, the way it is called a social organization of care that does not arrive at a system of care. So, the proposal we are working on and developing […] to create an ****articulated system ****of policies, actions, strategies, activities, programs, legislations, and measures aimed at guaranteeing care…” (Female, Civil Society Organizations, [05 C])*

Policies and programs at the organizational level fell into three main bins: healthcare, workplaces, and daycare. Baby-friendly hospital certification, which refers to an initiative by the WHO and UNICEF that encourages health facilities to better support breastfeeding, was mentioned by several respondents as being important for breastfeeding, but respondents suggested that their impact was limited by the relatively few hospitals participating and a failure to monitor compliance. Suggestions for potential policies in this area include encouraging more hospitals to become certified as baby-friendly and increasing monitoring and enforcement of compliance.

Respondents mentioned existing labor laws and guidelines pertaining to breastfeeding in the workplace, including Federal Labor Law which mandates break time for breastfeeding for the first six months and 12 weeks of guaranteed maternity leave for all postpartum workers. However, there was widespread recognition that these laws were not well understood nor enforced, particularly with respect to workers in the informal sector. In fact, several participants described a complete lack of federal labor laws to protect informally employed workers, apparently not realizing that such laws currently exist.

*“…But for me, in summary, informal workers in Mexico are those women who work, I mean, speaking specifically for women, in economic activities that are not regulated by the government, that ****do not have access to social security, labor rights***, *social protection, they do not have a job, maybe not so formal, or for a while, or they have these, like, super short contracts that do not guarantee them the duration or the seniority of these [the jobs]…” (Female, Civil Society Organization, [01 C])*

While informal employment was often defined as the lack of IMSS - the Mexican social security fund that provides paid maternity leave, health insurance, and free childcare, among other benefits - respondents described how IMSS could be expanded in ways that would support breastfeeding among women in informal employment. As of 2020, IMSS coverage is available to domestic workers, an overwhelmingly female workforce that is typically considered informally employed [31]. However, respondents noted that employers were not motivated to register their domestic workers. Potential policy expansions include increasing IMSS coverage to workers in other predominantly informal occupations and incentivizing employers to register their employees and contribute to IMSS. Finally, respondents mentioned the need for paid maternity (and paternity) leave for workers in the informal sector, which could be accomplished through expanding IMSS coverage, as previously discussed, or through something like a maternity cash transfer program, to be discussed further below.

Community level policies and programs included laws supporting the right to breastfeed in public, public lactation rooms, and state- or local-level nutrition education programs. Respondents also described breastfeeding promotion campaigns conducted by civil organizations, which respondents suggested could be complemented by governmental breastfeeding promotion campaigns, which were perceived as being able to extend their reach and impact.

Relatively few current or potential policies were aimed at the interpersonal level. These included current efforts to promote lactation support among partners and families, and a potential program to encourage father involvement in infant caregiving, including lactation support.

Finally, at the individual level, respondents described ongoing efforts to provide lactation consultation, education, and peer support, and to increase women’s knowledge of their rights with respect to lactation in the workplace. Several respondents described an existing cash transfer program for children of working mothers (Programa para el Bienestar de las Niñas y Niños, Hijos de Madres Trabajadoras, in Spanish) as valuable, but limited in its coverage.

*“…One of the eligibility criteria is that the mother does not have any access to childcare services, right? So, this is important because many of the mothers who work in the informal sector do not have any kind of social security. So, all these mothers can be beneficiaries of our program. And we give them [money] […] ****so that they can exercise their right and can even take their children to a childcare center.**** Or if they decide to pay someone to take care of them…” (Female, Government, [04G])*

According to information provided in a follow up email by the Ministry of Wellbeing, the cash transfer program–which provides MEX$1600 (about US$80) every two months to children ages 0–5 with single, working mothers–covered just under 300,000 children in 2022. By comparison, there are over 9 million children in this age range in Mexico. The potential policy expansion identified by respondents would be to increase coverage so that more families with young children could benefit from the program.

#### Theme 2: Research to enable policy/program formulation and implementation

While not a policy or program *per se*, research was described as enabling policy/program formation and implementation. For example, respondents described how research on the benefits of breastfeeding was shared with targeted decision-makers in order to impact policy conversations.

*“…And we also have different projects related to public policy recommendations to improve breastfeeding practices, in training also for health professionals to improve their skills for breastfeeding promotion in health centers. Evaluation of program policies. So it is like a diversity of projects and approaches to be able to study the different causes and ****provide the government with inputs so that they can implement policies or programs to improve breastfeeding practices***…*” (Female, Academia, [02 A])*

Respondents mentioned research into violations of the Code as critical in making the case for incorporating the Code into Mexican legislation. Research to estimate the costs of expanding paid maternity leave to workers in the informal sector has informed conversations about how best to achieve this goal. Lastly, the proposal for a National Care System was developed through research conducted by the Espinosa Yglesias Study Center (CEEY, for its Spanish name).

#### Theme 3: Breastfeeding as a right in policy discussions

Most respondents (11/14) used rights-based language when discussing breastfeeding and related policies/programs. Some respondents focused on the right to breastfeed while others emphasized the right to be breastfed, and still others discussed the role of the state in guaranteeing these rights.

*“…Ok, we have to first be clear that ****breastfeeding is a right***…*” (Male, Government, [06G])*

*“…Organizations have a very important role in guaranteeing the human rights of children, specifically, recognizing ****breastfeeding as a human right***, *as the United Nations has declared it to be…” (Female, Civil Society Organization, [03 C])*

Some respondents described a tension between the mother’s right to breastfeed and the baby’s right to be breastfed.

*“[H]ere there are two rights, the right of the mother to breastfeed and the right of the child to be breastfed. So, there is also ****a kind of tension between these two rights that are in collision, but by no fault of the mothers, but by fault of the space. ****I believe that the big infant feeding campaigns and the incorporation of women into the labor market somehow affected the visibility and recognition of the right to breastfeed. […] So women end up choosing […] but you must have a whole instrument […] you have to have a refrigerator […] conditions so that breast milk can be conserved. So, I think that finally ****a right that is basic and human is mixed up by conditions of privilege ****that are, well, belonging to a social class, having knowledge, knowing how to do it, and having minimum conditions for this…” (Female, Government, [01G])*

A related conflict also arose in discussions of caregiving (e.g., the rights of caregivers vs. the rights of those being cared for). One respondent described a strength of the National Caregiving System as its explicit focus on the rights of multiple actors, in contrast to individual policies or programs that may elevate the rights of one group over those of another.

#### Theme 4: Formal and informal workers as legally equivalent

Although most respondents acknowledged that a better understanding of informally employed workers in Mexico was needed, several emphasized the equivalence, rather than distinction, between formal and informal workers. In some cases, this was to stress that the Mexican Constitution applies equally to all kinds of workers, regardless of informality. Others stressed that to consider informal workers as distinct from formal workers is to validate this distinction and risk reinforcing two classes of workers.

*“There is an informal sector and it is an important sector because it is an important percentage of employment. But that doesn’t mean that the Federal Labor Law doesn’t apply to it, does it? So, yes, it is very important to keep that in mind that here ****in Mexico we do not see that difference***. *That is why many or all of the actions that are taken seek to dignify labor. And one thing to dignify work is precisely that they should be formal […] because that means all the protections, benefits, rights, and guarantees that a worker will have. […] Yes, it is a challenge. It has been a challenge and we have been seeking to formalize workers who are in the informal sector, as in parts, in steps…” (Female, Government, [03G])*

#### Theme 5: Need for institutionalization

Respondents highlighted the current challenge of temporary or one-off policies and programs, suggesting that, instead, these need to be enshrined in the constitution. This would prevent policies from being curtailed with the change of administration and ensures that government agencies have to account for them in their planning. Respondents also highlighted the necessity of having a dedicated budget.

*“…being reflected in effective public policies and then in budgets that cover these policies, in legal reform initiatives that support these actions and that are not just done for the photographs […] ****And the placement of these initiatives in corresponding legislation so that they really become a step towards guaranteeing rights***…*” (Female, Civil Society Organization, [03 C])*

#### Theme 6: Need for coordination among groups and institutions

Respondents mentioned the need for coordination across interventions and actions to protect, promote, and support breastfeeding, due to the absence of a public policy that integrates the efforts of different institutions, but also due to the lack of an expert group with representatives from different sectors (e.g., academia, international organizations, civil society, and government).

*“…There is an* ***absence of public policy*** *in the Mexican government that articulates breastfeeding efforts. So, the reality is that neither in the health sector nor in other sectors is there an articulation that allows us all to have clarity on how we join together with other, other allies.” (Male, Government, [06G])*

*“…We are missing a group of experts convened by a government institution. […] It was launched about two years ago and they have not met. So, in which different representatives from different sectors of the government, civil society, academia participate […] So, this is also lacking in Mexico, there is ****no institutional group on breastfeeding***, *it has been recommended on several occasions just to implement…” (Male, Civil Society Organization, [04 C])*

### Case study: maternity cash transfer seen as helpful, but not sufficient

Given the well-established connection between access to maternity leave and breastfeeding [9, 10], several recent studies have attempted to estimate the cost of expanding the duration of maternity leave for women in Mexico’s formal sector [28] and extending maternity leave through a cash transfer program to women in Mexico’s informal sector [21]. For this reason, we asked respondents specifically about the potential of a maternity cash transfer to promote, protect, and support breastfeeding among women in Mexico’s informal sector, whether it should be a conditional or unconditional transfer program, and where such a program could most effectively be administered within the federal government.

Overall, respondents had a positive response to the policy concept, saying that providing postpartum women with money would be helpful. However, respondents emphasized that a maternity cash transfer program on its own would be insufficient to promote, protect, and support breastfeeding because, as a short-term program, it would not address the underlying issue of informality. These women would still lack health insurance, job security, and employment benefits like paid time off after the transfer period ends.

*“…The fact that they are not in the formal sector is violating other rights. So, for example, if this program were part of the Ministry, if we were to give this money to working women in the informal sector, it would be like saying, continue in the informal sector, it is okay, we are going to give you this money for six months so that you can subsist, so that you can feed yourself and your baby, but after six months you continue in the informal sector. So, it would be like, it wouldn’t even be, like, the solution to the problem. […] So, yes*, ***giving them money during that period is going to help them […] but it will not solve their main problem***. *What is going to happen if that baby needs medical attention and she is not entitled to IMSS? […] She’s not going to have access to that free daycare no matter how much they pay for her six months of breastfeeding, then I think that would be ****not attacking the main problem***…*” (Female, Government, [03G])*

Participants mentioned the importance of accompanying this program with breastfeeding counseling, health and nutrition education, and installation of lactation rooms in public spaces. Some raised concerns about the ethics of conditioning financial support on breastfeeding outcomes without creating an environment that is fully supportive of a woman’s choice to breastfeed. Instead, they suggested that the cash transfer should be accompanied by, for example, breastfeeding education and related resources.

*“…For example, in rural areas, home visits at least the first week postpartum increase breastfeeding. So, if ****this transfer is accompanied by these counseling and breastfeeding interventions that have been successful***, *well, it has much more potential than just giving the transfer […] Also that they have access to lactation rooms, since they don’t have a workplace where they have lactation rooms. […] It would have to be like a ****comprehensive package for these women who normally do not have access to this type of benefits in their workplaces***…*” (Female, Academia, [02A])*

Others favored a conditional transfer out of concern that not doing so would reduce the likelihood that the policy’s objectives (e.g., increasing breastfeeding rates) would be achieved.

*“…What [the current government] has taught us is that if you change from conditional transfer to direct transfer, the public policy objectives are not necessarily met, because you are also talking about populations that have many constraints in their lives. So*, ***if you have additional cash and you do not tie it to anything, you are not necessarily going to use it to improve the nutrition of your children***. *You are not necessarily going to use it to improve their care, you are going to use it, literally Maslow’s pyramid, to fulfill your most pressing basic needs. So, I would condition the transfer…” (Female, Academia, [01A])*

The sole response to a maternity cash transfer program that was predominantly negative was rooted in the belief that working conditions had no impact on women’s decisions about whether and how long to breastfeed.

*“…Well, from my perspective I don’t think so, maybe this mechanism is not, maybe it is not the right one. ****No, because at the end of the day, each woman decides first, if she breastfeeds, and for how long***. *And you cannot condition her with a certain resource, tell her, no, we are going to pay you so much, but if you breastfeed for a year we are going to give you one part and if it is a year and a half, we will give you another part.” (Female, Government, [05G])*

## Discussion

Our study highlights the dearth of maternity protections that women employed in Mexico’s informal sector face. Few policies exist to promote, protect, and support breastfeeding among employed women, in general, but the economic vulnerability and challenging working conditions of women in informal employment exacerbates their situation. The lack of access to formal labor protections, such as paid maternity leave, creates a significant barrier to breastfeeding for women in the informal sector.

The study extends the body of literature about what works to promote, protect, and support breastfeeding among employed women in Mexico and beyond by focusing attention explicitly on women in the informal sector. In Mexico, 56% of women are informally employed, similar to other Latin American countries [17]. While some workers select informal work for its flexibility and other potential benefits, prior research suggests that informal work, particularly among women, is often involuntary and low paid [15]. Further, our study examines the perceptions of key informants who are engaged in developing and/or implementing programs and policies related to breastfeeding or informal employment in Mexico. Characterizing who they perceive as informal workers and what barriers they face is critical for understanding why certain policies have been successful in the past and which may hold promise going forward. Participants in our study described a wide range of barriers to breastfeeding that spanned individual to policy levels, suggesting that broad, systemic solutions might be feasible.

The discussion that follows highlights several key themes that emerged from the study’s findings and provides insights into potential policy solutions to address the barriers faced by women in the informal sector:

### Expansion of existing policies and programs

Respondents identified several potential policy expansions that build upon existing programs. For instance, fully incorporating the Code into Mexican legislation, coordinating lactation interventions and programs at the federal level, and expanding IMSS coverage to workers in predominantly informal occupations were suggested. Such expansions aim to strengthen the support system for breastfeeding women in the informal sector.

As with barriers to breastfeeding, many of the policies and programs that currently exist in Mexico apply broadly to all women (e.g., the Code, baby-friendly hospitals, laws pertaining to breastfeeding in public spaces) while others apply to all employed women (e.g., the Federal Labor Law). Relatively few policies and programs apply exclusively to women in informal employment (e.g., IMSS coverage of domestic workers, cash transfer program), yet these women are disproportionately impacted by weak implementation and enforcement of broader policy efforts [32]. As a result, recommendations for policy expansions often focused on ways to include informally employed women in programs that already exist for women in formal employment.

### Research for policy formulation and implementation

The importance of research in shaping policy conversations and supporting evidence-based decision-making was emphasized. Research on the benefits of lactation, violations of the Code, and the cost estimates of extending maternity leave through cash transfer programs were considered essential for developing effective policies.

### Rights-based language and gender equality

Many respondents framed breastfeeding as a right, both for the mother and the child. They emphasized the need to consider gender equality and caregiving responsibilities in policy discussions. Promoting breastfeeding should not only focus on individual choices but also address the structural constraints and societal norms that influence women’s decisions. At times, the rights of breastfeeding women were portrayed as being in conflict with the right to be breastfed. The intent of our study is not to prioritize one set of rights over another, but to illuminate the constraints and potential policies that could support breastfeeding goals. Breastfeeding policies must aim to generate a breastfeeding-friendly environment that makes it possible for women to decide on infant feeding practices, no matter their work conditions or type of work. There are several weaknesses in the system that are constraining women’s and children’s right to breastfeeding [33]. 

### Equivalence between formal and informal workers

Several respondents stressed the importance of treating formal and informal workers as legally equivalent. This perspective seeks to ensure that all workers, regardless of their employment status, are entitled to the same rights and protections and highlights the fact that informality itself is a foundational barrier. For the most part, respondents framed legal equivalence as the normative goal– that workers *should* be treated the same regardless of the formality of their work– while recognizing that informal workers currently face vastly different working conditions. This suggests that, while formalizing workers is a critical long-term goal, short-term strategies to support informally employed workers are needed. However, perceiving formal and informal workers as legally equivalent could also lead policymakers to not take action, or even to cut programs, if they believe that programs targeting informally employed workers are unnecessary.

### Institutionalization and coordination

Respondents highlighted the need for institutionalizing policies and programs to avoid them being short-lived or vulnerable to changes in administration. Furthermore, effective coordination among different groups and institutions was identified as crucial for the successful implementation of policies supporting lactation among women in the informal sector. Respondents mentioned examples of one-off partnerships across groups (i.e., government, civil society, international organizations, and academia), but emphasized the need for more coordination to increase the scope and impact of existing programs and policies. Such coordinated efforts currently do exist within some Mexican states (e.g., State of Mexico), but have not yet been translated to the national level.

### Maternity cash transfer

The study explored the potential of a maternity cash transfer program as a policy tool to support lactation among women in the informal sector. A vast body of literature links access to paid maternity leave with increased breastfeeding. But with more than half of Mexican women in informal employment, most women who give birth in Mexico lack access to paid maternity leave [17]. For this reason, recent studies have examined ways to extend maternity leave to women in Mexico’s informal sector through a cash transfer program [21]. While respondents generally saw this as a helpful measure, they also pointed out that it should be part of a comprehensive set of policies and programs. They emphasized the need to address the underlying issues faced by informal workers, such as lack of health insurance, job security, and employment benefits.

### Future directions

Caregiving and informal work are inextricably linked in the eyes of most respondents. The need to care for children often conflicts with formal jobs that lack flexibility. Even among highly educated women, respondents described a push from formal to informal employment among those with young children. Yet many proposed solutions aim to bring informal workers into the formal labor force, thus enabling them to take advantage of benefits that currently exist in the formal sector.

The National Care System (NCS) emerged from our interviews as having tremendous potential to protect, promote, and support lactation both through provisions directly related to lactation support, but also through encouraging formalization. The proposed NCS, which builds on similar systems across Latin America, seeks to enable social mobility through a system of support for those who provide and receive care [34, 35]. It proposes five action areas: a legal framework for the right to care; increase care infrastructure; improve the working conditions of domestic workers in order to increase their availability; promote the distribution of care across society; and expand social spending on care services [34]. As a system, the NCS has potential to address concerns about the lack of coordination across programs and institutions. Implementing the NCS in Mexico holds promise for supporting lactation across the economy.

Given the high percentage of women in Mexico who are informally employed, this sector demands increased and immediate attention in research and policy development. It is not sufficient to focus on how policies and programs to support women in formal paid employment impact breastfeeding outcomes when those policies and programs exclude more than half the female workforce. Instead, explicit attention must be placed on who is covered (and who is not) and how programs can be expanded to ensure benefits are distributed to those most in need. Mexico can look to many of its neighbors across Latin America to see how extending maternity cash benefits and other social protections to self-employed workers can be done [36]. 

These solutions, however, are temporary and are not without drawbacks. A rich literature has examined the labor market distortions that can arise from social protection programs, including cash transfers, that are linked to informality. Seguro Popular, for example, which offered virtually free health insurance to informally employed workers in Mexico, resulted in decreased formal employment and a reduction in the number of small and medium firms which are formally registered [37]. At the same time, the program filled a critical gap in health insurance coverage that affected the majority of Mexican workers and improved many health outcomes [38]. This predicament highlights the need to move towards decoupling social protection programs from employment and, instead, financing universal social protection through more sustainable general taxation [39]. This will become more and more pressing as non-standard forms of employment, including gig and app-based work, proliferate.

In the longer term, supporting the transition from informal to formal employment will increase social protection broadly for all workers. In addition to decreasing incentives for remaining in the informal sector through, for example, the expansion of universal and portable social protections, this will require the creation of more formal jobs and reducing the time and monetary costs associated with formalization [40, 41]. 

### Limitations

This study provides deep insight into the Mexican context, but findings can not be generalized to other countries. We spoke with 15 key informants representing 14 organizations that work closely with lactating and/or informally employed women. Inclusion of additional organizations, including those operating at the state and local levels, could impact our findings. Further, this study provides insights into how key informants from government, civil society, and academia perceive barriers to breastfeeding and the policies and programs that could potentially help informally employed women overcome them. However, in this study, the critical perspective of informally employed women, themselves, is limited. One organization representing informally employed women participated in our study, but three others did not respond to repeated requests for interviews. Building on this paper’s description of potential policies and programs to promote, protect, and support breastfeeding, future research should center the voices of women in informal employment to understand what they see as key barriers to breastfeeding and how they perceive these existing and potential policy and program supports.

## Conclusions

The study sheds light on the challenges and barriers that women in the informal sector face in maintaining breastfeeding practices and provides valuable insights into potential policy solutions. The discussion highlights the importance of a multi-level and multi-sectorial approach that involves policy expansions, research-based strategies, gender-sensitive language, equivalence of rights, institutionalization, and coordination among various stakeholders. Implementing these recommendations may contribute to promoting lactation and supporting the well-being of women in Mexico’s informal sector. However, it is essential to consider the context-specific challenges and opportunities when designing and implementing such policies to ensure their effectiveness and inclusiveness. Further research and collaboration among government, civil society, and academia will be critical to making progress toward social justice in lactation support for women in the informal sector.

### Electronic supplementary material

Below is the link to the electronic supplementary material.

Supplementary Material 1

Supplementary Material 2

Supplementary Material 3

Supplementary Material 4

Supplementary Material 5

## Data Availability

The datasets used and/or analyzed during the current study are available from the corresponding author on reasonable request.

## Footnotes

1 The results include descriptions of many programs, policies, and organizations. **Appendix table ****S1** maps the original Spanish name to an English translation and the acronym or shorthand we use to indicate each.
