# Supplementary material for: Breastfeeding among women employed in Mexico’s informal sector: strategies to overcome key barriers
Source: Int J Equity Health. 2024 Jul 23;23:144. doi: 10.1186/s12939-024-02147-x (PMC11264414; doi:10.1186/s12939-024-02147-x)
Supplement: Supplementary file 1 — Supplementary Material 1 [file 12939_2024_2147_MOESM1_ESM.docx]

**Additional file 1: Summary of each participating organization**

1. **Secretaría de Bienestar (Ministry of Welfare, in English)**

The Ministry is the government department in charge of social development efforts in Mexico. The Secretary of Welfare is a member of the Executive Cabinet. Between 1992 and 2018, the agency was known as the Ministry of Social Development (Secretaría de Desarrollo Social -SEDESOL-, in Spanish). The mission of the Ministry is to contribute to the establishment of a welfare state where people as subjects of rights, particularly historically vulnerable groups, improve their levels of welfare, inclusion, and equity during their life course, considering cultural, social, and territorial diversity, through the consolidation of comprehensive public policies, with sustainable development and productive inclusion. In terms of its general objectives, the following are included: I) Contribute to guaranteeing a basic set of human rights in an effective and progressive manner, starting with those most in need; II) Reduce socioeconomic inequality gaps between territories; III) Contribute to social welfare through sufficient income, promote food self-sufficiency, the reconstruction of the social fabric and generate the productive inclusion of farmers in rural areas to make the land productive; IV) Reduce the socioeconomic inequality gaps faced by historically excluded population groups, and V) Contribute to a culture of peace to reduce violence in communities and territories. One of its priority programs is the so-called "Program for the Welfare of Children of Working Mothers" (Programa para el Bienestar de las Niñas y Niños, Hijos de Madres Trabajadoras). The objective of this program is to contribute to improving the conditions of access to care and education, allowing the full exercise of the social rights of children, adolescents, and young people up to 23 years of age who are in a vulnerable situation due to the absence of one or both parents, through the delivery of financial support. As part of the program, children under 4 years of age receive bimonthly $1,600 MXN.

Link: <https://www.gob.mx/bienestar>

1. **Secretaría del Trabajo y Previsión Social -STPS- (Ministry of Labor and Social Welfare)**

The STPS in Mexico is a government ministry responsible for labor and employment-related matters, as well as social welfare policies and programs. Its primary role is to develop, implement, and enforce labor laws and regulations to protect the rights and promote the well-being of workers in Mexico. While their main areas of work may not directly revolve around breastfeeding, they play a role in promoting family-friendly workplace policies and supporting the rights of working mothers, which can indirectly impact breastfeeding practices. The following are some aspects related to their work:

1. Maternity Leave: The STPS establishes regulations and guidelines regarding maternity leave in Mexico. Maternity leave allows working mothers to have time off from work after giving birth, which can contribute to the initiation and continuation of breastfeeding.

2. Workplace Support: The Ministry promotes workplace policies and practices that support working mothers, such as providing breastfeeding breaks and designated spaces for expressing milk. These measures help create a supportive environment for breastfeeding mothers who return to work.

3. Labor Rights: The STPS ensures that working mothers are aware of their labor rights, including the right to breastfeeding breaks and accommodations. They may provide information and resources to employers and employees regarding the legal provisions and obligations related to breastfeeding support in the workplace.

4. Social Welfare Programs: While not directly related to breastfeeding, the Ministry's social welfare programs, such as conditional cash transfer programs and social security schemes, may indirectly support breastfeeding by improving the overall well-being of families and providing access to healthcare services.

Link: <https://www.gob.mx/stps/>

1. **Instituto Mexicano del Seguro Social -IMSS- (Mexican Institute of Social Security, in English)**

IMSS is a government institution responsible for providing social security services to workers and their families. It provides various types of social security services, including healthcare, occupational safety, and retirement pensions to Mexican workers and their families. The IMSS was created in 1943 and is considered one of the largest social security institutions in Latin America. IMSS operates as a decentralized public institution under the authority of the Secretaría de Salud (Ministry of Health, in English) in Mexico. IMSS is committed to promoting and supporting breastfeeding through various initiatives and programs:

I) Promotion of Breastfeeding: IMSS actively promotes breastfeeding as the optimal feeding method for infants. They raise awareness among expectant mothers, new mothers, and healthcare professionals about the benefits of breastfeeding and the importance of exclusive breastfeeding for the first six months of a baby's life.

II) Breastfeeding Education: IMSS provides breastfeeding education and counseling to mothers and families. They offer information on proper breastfeeding techniques, positioning, and overcoming common challenges. This education helps empower mothers with the knowledge and skills necessary for successful breastfeeding.

III) Training of Healthcare Professionals: IMSS conducts training programs and workshops for healthcare professionals, including doctors, nurses, and lactation consultants, to enhance their knowledge and skills in breastfeeding support and counseling. This training ensures that healthcare providers can effectively assist and guide mothers in their breastfeeding journey.

IV) Breastfeeding Support in Healthcare Facilities: IMSS healthcare facilities strive to create a breastfeeding-friendly environment. They encourage immediate skin-to-skin contact after birth, promote rooming-in (where babies stay with their mothers), and support on-demand breastfeeding. IMSS also ensures that healthcare facilities have designated spaces for breastfeeding or expressing milk to facilitate breastfeeding in public areas.

V) Support Groups and Counseling: IMSS organizes breastfeeding support groups where mothers can share their experiences, seek advice, and receive encouragement from other breastfeeding mothers and healthcare professionals. They also offer individual counseling to address specific breastfeeding challenges or concerns.

VI) Community Outreach: IMSS engages in community outreach programs to reach underserved populations and promote breastfeeding. They collaborate with local organizations, community leaders, and social workers to disseminate information, provide resources, and offer support to mothers and families.

Link: <http://www.imss.gob.mx/>

1. **Instituto Nacional de las Mujeres -INMujeres- (National Institute for Women, in English)**

The INMujeres in Mexico is a government institution that focuses on promoting gender equality, advancing women's rights, and eradicating gender-based discrimination and violence. It operates under the authority of the Secretaría de Gobernación (Ministry of Government, in English) in Mexico. The institute plays a crucial role in formulating and implementing public policies, programs, and actions to address gender inequality and empower women in various aspects of life. While its primary focus is on women's empowerment and gender issues, it also recognizes the importance of breastfeeding for maternal and child health through:

I) Policy Development: The institute collaborates with relevant stakeholders to develop policies and guidelines that support breastfeeding. They work towards ensuring that national policies promote and protect the rights of breastfeeding women, including measures to facilitate breastfeeding in public spaces and workplaces.

II) Advocacy and Awareness: The INMujeres engages in advocacy efforts to raise awareness about the benefits of breastfeeding and the importance of creating supportive environments for breastfeeding mothers. They work towards challenging social norms, combating stigma, and promoting positive attitudes towards breastfeeding.

III) Research and Data Collection: The institute supports and conducts research on breastfeeding practices and barriers in Mexico. This research helps identify specific challenges faced by breastfeeding women and informs evidence-based strategies and interventions to overcome these barriers.

IV) Capacity Building and Training: The institute provides training and capacity-building programs for healthcare professionals and community workers to enhance their knowledge and skills in breastfeeding support. This training aims to ensure that healthcare providers are equipped to provide accurate information, counseling, and assistance to breastfeeding women.

V) Collaboration and Partnerships: The Institute works collaboratively with other government agencies, civil society organizations, and international partners to promote breastfeeding. They engage in partnerships to implement joint initiatives, share best practices, and leverage resources to enhance breastfeeding support and promotion efforts.

VI) Breastfeeding and Work: The institute recognizes the challenges faced by working mothers in balancing breastfeeding and employment. They advocate for policies and programs that support breastfeeding in the workplace, such as flexible work arrangements, breastfeeding breaks, and designated breastfeeding spaces.

Link: <https://www.gob.mx/inmujeres>

1. **Centro Nacional de Equidad de Género y Salud Reproductiva – CNEGSR- (National Center for Gender Equity and Reproductive Health, in English)**

CNEGSR is the department of the Undersecretary for Prevention and Health Promotion and part of the Mexican Ministry of Health, responsible for national policies on family planning and contraception, sexual and reproductive health, cervical cancer, breast cancer, maternal and perinatal health, gender equality, and prevention of and attention to family violence. The Center focuses on promoting gender equity in health care, education, and social services, and works to ensure that all individuals, regardless of their gender, have access to quality health care services and information related to reproductive health. Some of the key functions and activities of the CNEGSR regarding breastfeeding are:

I) Policy Development: It participates in the development and implementation of policies and guidelines related to breastfeeding. They work with other government institutions, healthcare providers, and civil society organizations to ensure that breastfeeding is recognized and prioritized as an essential component of reproductive health and maternal and child well-being.

II) Training and Capacity Building: The center provides training and capacity-building initiatives for healthcare providers, particularly those working in reproductive health and maternity care. This includes training on breastfeeding counseling and support, promoting the importance of exclusive breastfeeding, and addressing common challenges faced by breastfeeding mothers.

III) Research and Data Collection: It conducts research and collects data related to breastfeeding practices and outcomes. This research helps inform policy decisions and program development, enabling evidence-based interventions to improve breastfeeding rates and support systems.

IV) Health Education and Communication: The center develops educational materials, campaigns, and communication strategies to raise awareness about the benefits of breastfeeding and provide accurate information to families. They target various audiences, including pregnant women, new mothers, families, and the general public, to promote positive attitudes towards breastfeeding and provide practical guidance on breastfeeding practices.

V) Collaboration and Partnerships: It collaborates with national and international organizations, healthcare institutions, civil society organizations, and professional associations to strengthen breastfeeding promotion and support. These collaborations aim to share knowledge, expertise, and resources to enhance breastfeeding programs and initiatives.

Link: <https://www.gob.mx/salud/cnegsr>

1. **Desarrollo Integral de la Familia -DIF- (Integral Family Development, in English)**

DIF is a decentralized public institution that focuses on social assistance and the well-being of vulnerable individuals and families. DIF operates at both the federal and municipal levels throughout the country. Each level has its own programs and initiatives tailored to the specific needs and circumstances of the local population. It was established in 1977 and operates under the umbrella of the Mexican government's Secretaría de Bienestar (Ministry of Welfare, in English). The primary objective of DIF is to provide social services, support, and protection to individuals and families facing various challenges, such as poverty, disability, domestic violence, neglect, and other forms of vulnerability. Some key aspects of DIF's work include social assistance, offering a range of social assistance programs and services to support families in need. The program “Atención Alimentaria en los Primeros Mil Días” (Nutritional Care in the First 1,000 Days, in English) is an initiative focused on providing nutritional support and comprehensive care to pregnant women, breastfeeding mothers, and children under two years of age, considering the critical period of the first 1,000 days of a child's life, which encompasses pregnancy to two years of age. The main objective of the program is to ensure adequate and nutritious feeding during this crucial period, promoting healthy growth and development of children and improving maternal health. The following are some key aspects of the program:

I) Food Supplements: The program provides specific food supplements for pregnant women, breastfeeding mothers, and children under two years of age. These supplements are designed to meet the additional nutritional needs during these critical stages of life.

II) Nutritional Education: The program offers nutritional education and guidance to pregnant women, breastfeeding mothers, and their families. Information is provided on the importance of a balanced diet, promoting exclusive breastfeeding for the first six months, and the appropriate introduction of complementary foods starting at six months of age.

III) Access to Health Services: The program aims to ensure access to quality health services, including prenatal and postnatal care, growth and development monitoring, and specialized medical care when needed. Linkages with health units are encouraged to ensure proper and timely monitoring of maternal and child health.

IV) Promotion of Breastfeeding: The program promotes and supports exclusive breastfeeding for the first six months of a child's life, as well as continued breastfeeding along with appropriate introduction of complementary foods from six months onwards. Counseling and support are provided to mothers to overcome common challenges, and the creation of breastfeeding-friendly environments in homes, communities, and health centers is encouraged.

V) Monitoring and Evaluation: The program conducts ongoing monitoring of program implementation and outcomes to assess its impact and make necessary adjustments. This involves data collection on the nutritional status of the women and children served, as well as the evaluation of the effectiveness of implemented interventions.

Link: <https://www.gob.mx/difnacional>

1. **El Pacto por la Primera Infancia (The Pact for Early Childhood, in English)**

El Pacto por la Primera Infancia in Mexico is a national initiative aimed at promoting the well-being and development of young children in the country. It is a joint effort between the Mexican government, civil society organizations, and the private sector to prioritize early childhood development as a key policy area. It was launched in 2019 and has three main objectives: I) To ensure that all children in Mexico have access to quality early childhood development services, including health care, education, nutrition, and protection from violence and exploitation; II) To promote the participation and engagement of families, communities, and civil society in early childhood development, and III) To strengthen public policies, programs, and systems related to early childhood development. Its work is based on scientific evidence and recognizes the critical importance of the first years of life in shaping a child's future development and well-being. This civil society organization with its own legal identity has 10 goals, the third of which is to increase breastfeeding in the first hour of life to 75% and exclusive breastfeeding in children under 6 months of age to 45%. One of its latest studies was related to the cost of implementing maternity leave in the formal and informal sectors.

Link: <https://www.pactoprimerainfancia.org.mx/>

1. **Infancia Plena**

Infancia Plena is a civil association founded in the State of Nuevo Leon, Mexico, in 2015, to offer support networks to women during pregnancy and breastfeeding. The association's mission is to protect the human rights of children, as well as to prevent and eradicate violence against women during pregnancy, breastfeeding, and child rearing. Its vision is to unite and coordinate the efforts and talents of individuals, organizations, companies, and agencies that wish to professionally contribute to the protection of early childhood. Its program called, “Lactancia materna, un acto de amor” (“Breastfeeding, an act of love”, in English) aims to increase the number of breastfed babies in Mexico, as well as the protection of breastfeeding as a human right. They have a virtual support group for women who are pregnant and breastfeeding. In this course they provide legal advice to pregnant and breastfeeding women regarding their labor rights.

Link: <https://infanciaplena.org/>

1. **Asociación de Consultores Certificados en Lactancia Materna -ACCLAM- (Association of Certified Breastfeeding Consultants, in English)**

ACCLAM A.C. is a Mexican organization whose members are Certified Lactation Consultants by the International Board of Lactation Consultant Examiners (IBCLC). This organization's vision is to transform health in Mexico through breastfeeding with the support of highly trained professionals. ACCLAM is engaged in several activities such as:

I) Lactation counseling: Providing specialized counseling and individualized support to breastfeeding mothers to overcome common challenges they may face during breastfeeding.

II) Education and training: Offering breastfeeding education and training programs for healthcare professionals, such as doctors, nurses, midwives, and lactation consultants, to enhance their knowledge and skills in breastfeeding promotion and support.

III) Breastfeeding support groups: Organizing and facilitating breastfeeding support groups where breastfeeding mothers can share their experiences, receive emotional support, and gain information about breastfeeding from other parents and breastfeeding experts.

IV) Advocacy and promotion: Engaging in advocacy and promotion activities for breastfeeding at the local or national level, working with other organizations and government entities to ensure that policies and programs supporting and promoting breastfeeding are implemented.

Link: <https://www.acclam.org.mx/>

1. **Centro de Estudios Espinosa Yglesias -CEEY- (Espinosa Yglesias Studies Center, in English)**

CEEY in Mexico is a non-profit research institution. It was founded in 2006 by Claudio X. González, a prominent Mexican businessman and philanthropist, with the goal of promoting research, analysis, and dialogue on key economic, social, and political issues facing Mexico and Latin America. The institution is named after two prominent Mexican economists, Agustín Espinosa and Manuel Yglesias. CEEY's mission is to generate high-quality research, analysis, and proposals that contribute to informed decision-making and public debate on issues related to economic and social development in Mexico and Latin America. The organization is committed to promoting evidence-based solutions to pressing issues such as poverty, inequality, corruption, education, health, and environmental sustainability. CEEY operates through several research programs, including the Economy and Development program, the Public Policy and Governance program, the Social Policy program, and the Education program. CEEY currently works on the "Sistema Nacional de Cuidados" (National Care System, in English). This is an initiative and public policy aimed at promoting and improving the provision of care for individuals in situations of dependency, particularly children, older adults, and people with disabilities. The main objective of the system is to recognize and value unpaid care work and strengthen the infrastructure and formal care services in the country. The initiative is based on the premise that care is a shared responsibility and should be addressed comprehensively by different stakeholders, including the government, civil society, and the private sector. Some relevant aspects of the system include:

I) Recognition of unpaid care work: The system seeks to value and recognize the unpaid care work traditionally carried out by women. This involves raising awareness of their contribution and promoting gender equity in the distribution of care responsibilities.

II) Development of policies and programs: The system entails the development of specific policies and programs to improve the quality and accessibility of care services and promote the reconciliation of paid work and care responsibilities.

III) Care infrastructure: The system aims to strengthen the care infrastructure, including the creation of childcare centers, day care centers for older adults, and specialized centers for people with disabilities. This facilitates access to quality services and provides support to families in need of care for their loved ones.

IV) Training and capacity building: The system includes training and capacity-building programs for care providers, such as social workers, nurses, caregivers, and early childhood education personnel. This ensures that care services are provided by trained and qualified personnel.

V) Interinstitutional coordination: It promotes coordination among different governmental institutions and agencies to improve the efficiency and effectiveness of care services. This involves collaboration between sectors such as health, education, labor, and social development, among others.

Link: <https://ceey.org.mx/>

1. **Women in Informal Employment: Globalizing and Organizing -WIEGO-**

WIEGO is a global network and research-policy-action organization that focuses on promoting the rights and improving the conditions of women working in the informal economy. While WIEGO operates on a global scale, it also has initiatives and activities in Mexico. In Mexico, WIEGO collaborates with local partners and organizations to address the challenges faced by women in informal employment. The organization aims to increase the visibility and recognition of women's informal work, advocate for their rights, and support their economic empowerment. Some of the key areas of WIEGO’s work in Mexico include:

I) Research and Data Collection: WIEGO conducts research and collects data on the informal economy in Mexico, with a particular focus on women's participation and experiences. This research helps to inform evidence-based policies and interventions that address the specific needs and challenges faced by women in informal employment.

II) Policy Advocacy: WIEGO engages in policy advocacy at the national and local levels to promote the rights of women in the informal economy. The organization works to raise awareness among policymakers and the public about the contributions and needs of women in the informal sector, and advocates for policies and programs that improve their working conditions, access to social protection, and economic opportunities.

III) Capacity Building and Organizing: WIEGO supports capacity building initiatives for women in the informal economy, including training programs and workshops on entrepreneurship, financial literacy, and workers' rights. The organization also promotes the organizing of women in informal sectors to strengthen their collective voice and bargaining power.

IV) Knowledge Sharing and Networking: WIEGO facilitates knowledge sharing and networking among organizations and individuals working on issues related to the informal economy in Mexico. The organization organizes conferences, workshops, and forums to bring together stakeholders, share best practices, and foster collaboration.

Through its initiatives in Mexico, WIEGO aims to contribute to the improvement of working conditions, livelihoods, and empowerment of women in the informal economy. It strives to create an enabling environment that recognizes and values the contributions of women in informal employment and promotes their social and economic well-being.

Link: <https://www.wiego.org/es>

1. **UNICEF Mexico**

The United Nations Children's Fund (UNICEF) is the United Nations agency focused on promoting the rights and well-being of all children and adolescents in Mexico and around the world. It was created in 1946 to provide urgent assistance to child victims of war. UNICEF has been working in Mexico since 1954 in coordination with the government, civil society organizations and the private sector, and guided by the United Nations Convention on the Rights of the Child to ensure that all children and adolescents are guaranteed their rights. The Convention was signed by Mexico, which is committed to implementing it in the country. In general, UNICEF works towards ensuring that every child in the country can enjoy their rights, have access to essential services, and achieve their full potential, particularly those who are most vulnerable or marginalized. The following are some activities carried out by UNICEF Mexico regarding breastfeeding:

I) Advocacy and Policy Support: UNICEF works with governments and partners to advocate for policies and legislation that protect, promote, and support breastfeeding. This includes encouraging countries to adopt and implement the World Health Organization's (WHO) International Code of Marketing of Breast-milk Substitutes, which regulates the marketing of breast-milk substitutes and aims to protect and promote breastfeeding.

II) Education and Awareness: UNICEF supports breastfeeding education programs targeting families, healthcare providers, and communities. They provide information on the benefits of breastfeeding, proper breastfeeding techniques, and the importance of exclusive breastfeeding during the first six months of a child's life.

III) Baby-Friendly Hospitals and Health Facilities: UNICEF works to establish and support "Baby-Friendly Hospitals" and health facilities. These facilities implement the Ten Steps to Successful Breastfeeding, a set of evidence-based practices to support breastfeeding initiation and continuation. They also provide training and support to healthcare providers on breastfeeding counseling and support.

IV) Community Support: UNICEF promotes community-based support for breastfeeding mothers through the establishment of support groups, mother-to-mother networks, and community health worker programs. These initiatives help create an enabling environment for breastfeeding, provide peer support, and address common challenges faced by breastfeeding mothers.

V) Emergency Response: In the event of emergencies, such as natural disasters or conflicts, UNICEF works to ensure the protection and support of breastfeeding in affected communities. They provide breastfeeding support services, establish safe spaces for mothers and infants, and supply essential breastfeeding equipment.

Link: <https://www.unicef.org/mexico/>

1. **Universidad Iberoamericana -IBERO- (Iberoamerican University, in English)**

IBERO is a private university located in Mexico City, Mexico. It is one of the most prestigious and recognized institutions of higher education in the country. The university was founded in 1943 and operates under the sponsorship of the Society of Jesus (Jesuits), an influential Catholic religious order known for its commitment to education and social justice. IBERO, with its diverse faculties and research centers, may undertake research projects related to breastfeeding and maternal-child health within various disciplines, such as medicine, public health, nursing, psychology, social sciences, and nutrition. These projects aim to generate knowledge, promote evidence-based practices, and address issues surrounding breastfeeding. IBERO's research has contributed to analyzing existing policies related to breastfeeding, identifying gaps, and advocating for evidence-based policy changes. The results have been able to inform policy makers about the importance of breastfeeding and help shape public health strategies and interventions to support breastfeeding practices.

Link: <https://ibero.mx/>

1. **Instituto Nacional de Salud Pública -INSP- (National Institute of Public Health, in English)**

The INSP in Mexico is a prominent governmental institution dedicated to advancing public health research, education, and practice in the country. It focuses on generating knowledge, informing policies, and promoting evidence-based practices to improve the health of the population. The INSP’s work is multidisciplinary and encompasses various fields of public health. Through research, training, policy development, and partnerships, the institute contributes to improving the health and well-being of the population in Mexico. The following are some key aspects of the institute's work:

I) Research: The INSP conducts extensive research in various areas of public health, including epidemiology, health systems, environmental health, infectious diseases, chronic diseases, and nutrition. The INSP conducts research to generate evidence on breastfeeding practices, determinants, and impacts in Mexico. This research helps inform policies and interventions aimed at improving breastfeeding rates and outcomes.

II) Policy Development and Consultation: INSP plays a crucial role in shaping public health policies and strategies in Mexico related to breastfeeding. This includes guidelines for healthcare providers, recommendations for maternity leave and workplace support, and regulations regarding the marketing of breast milk substitutes.

III) Surveillance and Monitoring: INSP participates in monitoring and surveillance systems to assess breastfeeding practices and trends in Mexico. This information helps identify areas that require intervention and evaluate the effectiveness of breastfeeding promotion programs.

Link: <https://www.insp.mx/>
