# Supplementary material for: Breastfeeding among women employed in Mexico’s informal sector: strategies to overcome key barriers
Source: Int J Equity Health. 2024 Jul 23;23:144. doi: 10.1186/s12939-024-02147-x (PMC11264414; doi:10.1186/s12939-024-02147-x)
Supplement: Supplementary file 2 — Supplementary Material 2 [file 12939_2024_2147_MOESM2_ESM.docx]

Interview Guide

Version: December 5, 2022 (English)

The purpose of this study is to document existing labor policies and programs that promote, protect, and support lactation among informally employed women in Mexico. Specifically, by the end of this project, we hope to characterize informally employed women in Mexico; identify labor policies and programs - existing and potential - to support lactation among informally employed women in Mexico; and document the barriers to uptake of existing and potential labor policies and programs to support lactation among informally employed women in Mexico.

We have invited you to participate in this study because of your organization’s expertise and interest in using policies or programs to improve breastfeeding practices and/or in improving the health of informally employed women in Mexico.

Before we begin, I would like to ask whether you have any questions about the description of the study that I previously shared with you, the process for this interview, or any other question. *[Leave time for the interviewee to ask questions.]*

Thank you for agreeing to participate in this study. Lastly, I’d like to inform you that we are going to record the interview. Is that ok? *[Leave time for the interviewee to accept or reject recording of the interview.]*

*[If the interviewee is ok with audio-recording, continue:]* Thank you, Julia will begin recording the interview now.

Briefly describe your organization and the ways it relates to either lactation or informally-employed women in Mexico.

Briefly describe your role in the organization.

Who makes up the population of informally-employed women in Mexico?

What do you think are the main barriers to lactation among informally-employed women?

What policies and programs are currently available to protect, promote, and/or support lactation among informally-employed women?

What kinds of policies that may not yet exist do you think could help to protect, promote, and support lactation among informally-employed women in Mexico?

One proposal for supporting breastfeeding in this population is to extend paid maternity leave to informally employed women through a non-contributory cash transfer program. Do you think this kind of program would protect, promote, and/or support breastfeeding among informally-employed women? Do you think this kind of program would be feasible to implement in Mexico?

What are barriers to formalization among informally-employed women in Mexico?

What is the role of the government/NGOs/your organization in supporting lactation among informally-employed women?

Is there anything else you want to share that I haven’t asked you about today?

Thank you for taking time to speak with us today. We appreciate your contributions.
