# Supplementary material for: Breastfeeding among women employed in Mexico’s informal sector: strategies to overcome key barriers
Source: Int J Equity Health. 2024 Jul 23;23:144. doi: 10.1186/s12939-024-02147-x (PMC11264414; doi:10.1186/s12939-024-02147-x)
Supplement: Supplementary file 4 — Supplementary Material 4 [file 12939_2024_2147_MOESM4_ESM.docx]

**Additional file 4.** Representative quotes from in-depth interviews based on the Socio-Ecological Model (SEM) (*n* = 15).

| **Category** | **Original Quotations** | **English Translated Quotation** |
| --- | --- | --- |
| Informal Job Characteristics | *“... En México la informalidad para mí tiene encubre una altísima situación de precariedad de los trabajadores. En realidad es trabajo precario, más que trabajo informal. ¿Qué quiere decir con eso? Que pareciera que cuando decimos informal […] pareciera que lo único que falta es el contrato. Cuando en realidad lo que está pasando en la informalidad es una altísima precariedad. Y entonces esta precariedad lo que produce es muchísima desigualdad en relación a los otros trabajadores, que son formales, pero también está muy atravesada por el orden de género. En México, tiene muy baja incorporación de las mujeres al mercado laboral formal…” (Female, Government, [01G])* | *“...In Mexico, I believe that informality masks a very high level of precarity among workers. In reality, it is precarious work, more than informal work. What do I mean by that? It would seem that when we say informal, it would seem that the only thing missing is a contract. When in fact what is happening in informality is a very high level of precarity. And then this precarity produces a great deal of inequality in relation to other workers, who are formal, but it is also very much affected by the gender order. In Mexico, there is a very low incorporation of women into the formal labor market…” (Female, Government, [01G])* |
| Barriers to breastfeeding among women employed in Mexico’s informal sector | *“...Creo que la desinformación es una barrera transversal, porque cuando quien legisla no tiene información, quien hace presupuestos no tiene información. Quien hace políticas públicas no tiene información. Quien te topa en la línea del metro no tiene información. Tu derecho a la lactancia se va a ver pisoteado al por mayor…” (Female, Civil Society Organization, [03C])*  *“...Y la otra cosa, que también me parece como muy absurda de parte del gobierno…o sea como que dice en la informalidad no hay derechos. Eso jurídicamente es falso, porque si uno revisa el marco jurídico de los derechos humanos, cuando se habla del derecho al trabajo, se habla del derecho al trabajo a trabajar. No dice formalidad o informalidad, no dice a todos los que trabajan en la formalidad van a tener estos derechos. No dice nunca eso. O sea, las personas que trabajen tienen estos derechos. Entonces, lo que corresponde al Estado es extender los derechos de las personas trabajadoras a la informalidad también…” (Female, Civil society organizations, [06C])*  *“... Entonces para mí las coloca como en una vulnerabilidad, sobre todo por el tema de los recursos económicos. Y eso desencadenaría para mí un montón de cosas como el estrés y entonces problemas de salud mental. Y muchísimas cosas…” (Female, Civil society organizations, [01C])*  *“... No sé, ese puede ser un factor el que no tengan, por ejemplo, las que están en el sector informal no van a tener a lo mejor el mismo acceso que algunas mujeres del sector formal, no todas, a salas de lactar o extractores de leche. Que algunas mujeres del sector formal sí tienen, no todas, pero algunas, de hecho las del sector informal es mucho más complicado que los tengan [salas de lactar o extractores de leche]…” (Female, Academia, [02A])*  *“... Otra barrera es que claro, los utensilios para guardar, almacenar, transportar la leche materna, pues también tienen un costo que no siempre cuentan con este las mujeres…” (Male, International Organization, [04C])* | *“... I believe that misinformation is a cross-cutting barrier, because when legislators do not have information, when budget makers do not have information. Those who make public policies have no information. Whoever you meet in the subway line does not have information. Your right to breastfeeding is going to be wholesale trampled…” (Female, Civil Society Organization, [03C])*  *"...And the other thing, which also seems to me to be nonsense on the part of the government, is to say that in informality there are no rights. This is legally false because if one reviews the legal framework of human rights when it talks about the right to work, it talks about the right to work. It doesn’t say formality or informality, it doesn’t say that all those who work in formality will have these rights. It never says that. In other words, people who work have these rights. So, what corresponds to the State is to extend the rights of working people to the informal sector as well..." (Female, Civil society organizations, [06C]).*  *"... So for me it places them in a state of vulnerability, especially because of the issue of economic resources. And that would trigger for me a lot of things like stress and then mental health problems. And a lot of things..." (Female, Civil society organizations, [01C])*  *"... I don't know, that could be a factor that they do not have, for example, those in the informal sector may not have the same access as some women in the formal sector, not all of them, to lactation rooms or breast pumps. Some women in the formal sector do have them, not all, but some, in fact for those in the informal sector it is much more complicated for them to have them [lactation rooms or breast pumps]..." (Female, Academia, [02A]).*  *"... Another barrier is that, of course, the equipment for storing and transporting breastmilk also has a cost that women are no always able to pay..." (Male, International Organization, [04C])* |
| Barriers to formalization for women | *“...En México una mujer puede dedicar hasta 40 horas a la semana a actividades no remuneradas dentro del hogar. En tareas domésticas, cuidado de menores, personas enfermas, adultos mayores. Y esto equivale, pues, casi a un trabajo de tiempo completo…” (Male, Government, [06G])*  *“...¿Cuáles son las barreras para reducir la informalidad? Pues creo que todavía el hecho de que muchas de nosotras las mujeres tengamos justamente este prejuicio o esta educación de que la mujer todavía es mucho de la casa, de la atención de la familia, de la atención exclusiva de los niños. Es todavía un prejuicio que creo que no favorece el hecho de la incorporación de la mujer plena al trabajo. Todavía hay mucha, muchas barreras socio-culturales e incluso todavía en el área metropolitana en donde, pues si no es posible que ya ella se puedan reincorporar y terminan entonces ejerciendo un empleo informal. Me dedico a vender por catálogo, me dedico a vender ropa y zapatos, me dedico a poner una papelería, algo en donde yo pueda compaginar más el el hecho de ser cuidadora de los niños o tal vez cuidadora, incluso de otras personas, de mis papás o de personas mayores, de alguna persona enferma…” (Female, Government, [02G])*  *“...¿Y si son madres responsables de familia, madre o jefas de familia, pues no pueden tomar un trabajo formal porque es de 9 a 5 si les va bien o de 9 a 7 y ellas no tienen ayuda, no? Entonces eso las empuja a trabajar en sectores informales que a lo mejor desde chicas no trabajaron ahí, no fueron jornaleras agrícolas, por ejemplo, pero sí fueron madres solteras que necesitan hacerse cargo de sus hijos, que no tienen ayuda de ningún tipo y que necesitan ciertos horarios y cierta flexibilidad que en el sector formal no tienen…”* (*Female, Government, [03G])*  *“... Su nivel educativo no permite que se incorpore a la formalidad…”* (*Female, Academia, [01A])*  *“...Yo te apuesto que si mañana deciden igual todas estas personas, despertar e ir a buscar un trabajo formal, no alcanzarían los trabajos formales para estas personas. Es decir, no existe la oferta laboral en la formalidad para todas las personas que trabajan en el empleo informal…”* (*Female, Civil Society Organization, [*06C*])*  *“...No es que un trabajador o una trabajadora un día se despierte y diga ah, hoy voy a tener un trabajo formal y va y lo busca y lo encuentra. No funciona así…”* (*Female, Civil Society Organization, [*06C*])*  *“...Y esta es otra barrera, que no hay suficiente capacitación para mujeres que actualmente se emplean en el empleo informal que sea accesible a ellas, que se ajuste a sus necesidades, que permita mejorar sus habilidades, que les den más herramientas para incorporarse al empleo formal...”* (*Female, Academia, [01A])* | *"...In Mexico, a woman can dedicate up to 40 hours a week to unpaid activities within the home. In domestic chores, caring for children, sick or elderly people. And this is almost equivalent to a full-time job..." (Male, Government, [06G])*  *"...What are the barriers to reducing informality? Well, I think that the fact that many of us women still have this prejudice or this upbringing that women are still very much in charge of the house, of the care, of the family, of the exclusive care of the children. It is still a prejudice that I believe does not favor the full incorporation of women in the workplace. There are still many, many socio-cultural barriers, and even in the metropolitan area where, well, if it is not possible for them to reincorporate themselves, they end up working in informal jobs. I sell by catalog, I sell clothes and shoes, I start a stationery store, something where I can combine being a caregiver for the children or maybe even a caregiver for other people, for my parents or for the elderly, for someone who is ill..." (Female, Government, [02G])*  *"...And if they are mothers responsible for their families, mothers or heads of household, they cannot take a formal job because it is from 9 to 5 if all goes well or from 9 to 7 and they have no help, right? So that pushes them to work in informal sectors that maybe they haven’t worked in all their lives, they were not agricultural day laborers, for example, but they were single mothers who need to take care of their children, who do not have any kind of help and who need certain schedules and flexibility that they do not have in the formal sector..." (Female, Government, [03G])*  *"... Their educational level does not allow them to join formality..." (Female, Academia, [01A])*  *"...I bet you that if tomorrow all these people decided to wake up and look for a formal job, there would not be enough formal jobs for these people. That is to say, there are not enough formal job offers for all the people who work in informal employment..." (Female, Civil Society Organization, [06C])*  *"...It is not that a worker wakes up one day and says: "Today I am going to have a formal job" and goes and looks for it and finds it. It doesn't work like that..." (Female, Civil Society Organization, [06C]).*  *"...And this is another barrier, that there is not enough training for women who are currently employed in informal employment that is accessible to them, that fits their needs, that allows them to improve their skills, that gives them more tools to join formal employment..." (Female, Academia, [01A])* |
| Potential policies as expansions of current policies/programs | *“...Y a partir de esta idea general, lo que hoy tenemos en México es un conjunto de acciones de prácticas desarticuladas, orientadas al cuidado, como son, por ejemplo las estancias infantiles, como son las escuelas de tiempo completo para el cuidado de niñas, niños en edad escolar. Algunas otras políticas o medidas que se han establecido en la ley, como por ejemplo las unidades de cuidados paliativos para personas en condiciones de enfermedades terminales. Algunas acciones aisladas como la integración de un padrón de personas o un registro de personas con discapacidad. Este y bueno, todo eso, lo que la manera en como se se denomina es una organización social del cuidados que no llega a ser un sistema de cuidados. Entonces, la propuesta que nosotros estamos trabajando y desarrollando [...] conformar un sistema articulado de políticas, acciones, estrategias, actividades, programas, legislación y medidas orientadas a garantizar el cuidado…” (Female, Civil Society Organizations, [05C])*  *“...Además no tienen acceso a ningún tipo de políticas que garanticen la protección de la lactancia materna como derecho humano…” (Female, Civil Society Organization, [03C])*  *“...Para mí un trabajo informal es aquello que no tiene justo eso, la formalidad de un contrato laboral que le pueda aplicar todos los derechos y todos los deberes que pudiera tener dentro de una empresa o un espacio donde labore y todas las prestaciones que se le tengan que brindar, que por lo menos deben de ser las establecidas dentro de la ley. Si no tiene ese contrato, pues cualquier otra mujer que se dedique a algo que no lo tenga, pues cae en el área de trabajadora informal. Ya sea que se dedique al comercio, a hacer las casas, a cuidar niños, a hacer limpieza, en seguridad o a lo que se dedique, si no hay un contrato de por medio que le dé esta derechohabiencia, pues entonces es una trabajadora informal…” (Female, Government, [05G])*  *“...Pero para mí, en resumen, las trabajadoras informales en México son aquellas mujeres que trabajan, digo, hablando específicamente para las mujeres, en actividades económicas que no están reguladas por el gobierno, que no tienen acceso a seguridad social, derechos laborales, protección social, no tienen un trabajo, tal vez tan formal, o por un tiempo, o tienen estos contratos como súper cortos que no les garantizan la duración o la antigüedad de estos [los trabajos]…” (Female, Civil Society Organization, [01C])*  *“...Y aquí es bien importante porque se les da un apoyo social. ¿Y uno de los criterios de elegibilidad es que la madre no cuente con algún servicio de cuidado o atención infantil, no? Entonces este esto pues es importante porque muchas de las de las madres que trabajan en el sector informal, pues no cuentan con ningún tipo de seguridad social. Entonces todas esas madres pueden ser beneficiarias de nuestro programa. Y se les entrega [dinero] [...] para que ellas puedan ejercer su derecho y puedan incluso llevar a sus hijos a alguna estancia infantil. O si deciden pues pagarle a alguien para que lo cuide…” (Female, Government, [04G])* | *"...And from this general idea, what we have in Mexico today is a set of actions of disjointed practices, oriented towards care, such as, for example, childcare centers, such as full-time schools for the care of school-aged children. Some other policies or measures that have been established in the law, such as palliative care units for people with terminal illnesses. Some isolated actions such as the integration of a census of persons or a registry of persons with disabilities. This, well, all this, the way it is called a social organization of care that does not arrive at a system of care. So, the proposal we are working on and developing [...] to create an articulated system of policies, actions, strategies, activities, programs, legislation and measures aimed at guaranteeing care..." (Female, Civil Society Organizations, [05C]).*  *"...Besides, they do not have access to any kind of policies that guarantee the protection of breastfeeding as a human right..." (Female, Civil Society Organization, [03C])*  *"...For me, an informal job is one which does not have just that, the formality of a labor contract that can apply all the rights and duties that you could have within a company or a space where you work and all the benefits that must be provided, which should at least be those established by law. If she does not have this contract, well, any other woman who works in something that does not have [a contract] falls into the area of informal worker. Whether she works in commerce, housekeeping, childcare, cleaning, security or whatever she does, if there is no contract that gives her this right, then she is an informal worker..." (Female, Government, [05G])*  *"...But for me, in summary, informal workers in Mexico are those women who work, I mean, speaking specifically for women, in economic activities that are not regulated by the government, that do not have access to social security, labor rights, social protection, they do not have a job, maybe not so formal, or for a while, or they have these, like, super short contracts that do not guarantee them the duration or the seniority of these [the jobs]..." (Female, Civil Society Organization, [01C])*  *"...And here it is important because it gives them social support. And one of the eligibility criteria is that the mother does not have any access to child care services, right? So this is important because many of the mothers who work in the informal sector do not have any kind of social security. So all these mothers can be beneficiaries of our program. And we give them [money] [...] so that they can exercise their right and can even take their children to a childcare center. Or if they decide to pay someone to take care of them..." (Female, Government, [04G]).* |
| Research to enable policy/program formation and implementation | *“... Y tenemos también diferentes proyectos relacionados con recomendaciones de política pública para mejorar las prácticas de lactancia, en capacitación, también a profesionales de la salud para mejorar sus habilidades para la promoción de lactancia materna en los centros de salud, evaluación de políticas de programas. Entonces es como una diversidad de de proyectos y de enfoques para poder estudiar las diferentes causas y darle al gobierno insumos para que puedan implementar políticas o programas que mejoren las prácticas de lactancia…” (Female, Academia, [02A])* | *"... And we also have different projects related to public policy recommendations to improve breastfeeding practices, in training also for health professionals to improve their skills for breastfeeding promotion in health centers. Evaluation of program policies. So it is like a diversity of projects and approaches to be able to study the different causes and provide the government with inputs so that they can implement policies or programs to improve breastfeeding practices..." (Female, Academia, [02A]).* |
| Breastfeeding as a right in policy discussions | *“...Ok, tenemos que primero tener claro que la lactancia materna es un derecho...” (Male, Government, [06G])*  *“...Las organizaciones tenemos un papel muy importante en la garantía de los derechos humanos de la niñez, específicamente hablando en el tema de reconocer la lactancia materna como un derecho humano, tal como las Naciones Unidas lo han declarado así…” (Female, Civil Society Organization, [03C])*  *“...Porque no solamente hay, aquí hay dos derechos, el derecho a la madre amamantar y el derecho de la criatura a ser amamantado. Entonces también hay como como una tensión entre esos dos derechos que están en colisión, pero no por culpa de la madre, sino por culpa del espacio. Yo creo que las grandes campañas de alimentación y de incorporación de las mujeres al mercado laboral de alguna manera afectaron la visibilización y el reconocimiento del derecho de la lactancia materna. [...] Entonces las mujeres terminan eligiendo [...] pero hay que tener todo un instrumento, hay que tener una ingeniería de posibilidades. [...] hay que tener un refrigerador, hay que tener condiciones también para que la leche materna pueda ser conservada. Entonces creo que finalmente un derecho que es básico y humano termina atravesado por el privilegio, termina siendo atravesado por condiciones de privilegio que son, pues el pertenecer a una clase social, el tener un conocimiento, el saber cómo hacerlo y el también el tener condiciones mínimas para esto…” (Female, Government, [01G])* | *"...Ok, we have to first be clear that breastfeeding is a right..." (Male, Government, [06G])*  *"...Organizations have a very important role in guaranteeing the human rights of children, specifically, recognizing breastfeeding as a human right, as the United Nations has declared it to be..." (Female, Civil Society Organization, [03C])*  *"...Because there are not only, here there are two rights, the right of the mother to breastfeed and the right of the child to be breastfed. So there is also a kind of tension between these two rights that are in collision, but by no fault of the mother’s, but by fault of the space. I believe that the big infant feeding campaigns and the incorporation of women into the labor market somehow affected the visibility and recognition of the right to breastfeed. [...] So women end up choosing [...] but you have to have a whole instrument, you have to have well-structured options. [...] you have to have a refrigerator, you also have to have conditions so that breast milk can be conserved. So I think that finally a right that is basic and human is mixed up by conditions of privilege that are, well, belonging to a social class, having knowledge, knowing how to do it, and also having minimum conditions for this..." (Female, Government, [01G]).* |
| Formal and informal workers as legally equivalent | *“... La Ley no se hace esta distinción de a quien le aplique, que si al sector formal e informal le aplican unas cosas o no. Existe el sector informal y es un sector importante porque es un porcentaje importante del empleo. ¿Pero eso no significa que no les aplique la Ley Federal del trabajo, no? Entonces sí es muy importante tener eso en cuenta que aquí en México no vemos esa diferencia. Por eso muchos o todas las acciones que se hacen buscan dignificar el trabajo. Y una cosa para dignificar el trabajo es precisamente que sean formales, que sean empleos formales, porque eso habla de todas las protecciones, prestaciones, derechos y garantías que va a tener un trabajador. [...] Sí, es un reto. Ha sido un reto y que lo hemos estado buscando formalizar a los trabajadores que están en el sector informal, como en partes, en pasos…” (Female, Government, [03G])* | *"... The Law does not make this distinction as to who it applies to, whether certain things apply to the formal or informal sector. There is an informal sector and it is an important sector because it is an important percentage of employment. But that doesn’t mean that the Federal Labor Law doesn’t apply to it, does it? So, yes, it is very important to keep that in mind that here in Mexico we do not see that difference. That is why many or all of the actions that are taken seek to dignify labor. And one thing to dignify work is precisely that they should be formal, that they should be formal employees, because that means all the protections, benefits, rights, and guarantees that a worker will have. [..] Yes, it is a challenge. It has been a challenge and we have been seeking to formalize workers who are in the informal sector, as in parts, in steps…" (Female, Government, [03G])* |
| Need for institutionalization | *“...Entonces, esta desinformación de la comunidad desde una visión ecológica impide que las mujeres sean valoradas en su decisión de amamantar a sus hijos e hijas e impide que eso se refleje en políticas públicas efectivas y después en presupuestos que cobijen esas políticas, en iniciativas de reforma de ley que tengan un respaldo a esas acciones y que si se hacen, se dejen de hacer para la fotografía [...] Y la colocación de estas iniciativas en la legislaciones correspondientes para que realmente sean, pues, un paso a la garantía de derechos…” (Female, Civil Society Organization, [03C])* | *"...So, this misinformation of the community from an ecological point of view prevents women from being valued in their decision to breastfeed their children and prevents this from being reflected in effective public policies and then in budgets that cover these policies, in legal reform initiatives that support these actions and that are not just done for the photographs [...] And the placement of these initiatives in corresponding legislation so that they really become a step towards guaranteeing rights..." (Female, Civil Society Organization, [03C])* |
| Need for coordination among groups and institutions | *“...Algo que quiero compartirte…es que hay una ausencia en el gobierno de México de política pública que articule los esfuerzos de lactancia materna. Entonces, la realidad es que ni en el sector salud ni en otros sectores hay una articulación que nos permita tener a todos claridad en cómo sumamos con otros, otros aliados… Entonces tenemos una brecha y una ausencia de política pública que nos ayude a coordinar esos procesos de articulación...” (Male, Government, [06G])*  *“...Falta como un grupo de expertos convocado por una institución de gobierno, que en principio existe pero nunca convocan. Se lanzó hace como dos años y no se han reunido. Entonces, en el que participen diferentes representantes de los diferentes sectores del gobierno, sociedad civil, la academia, para que justamente se puedan discutir estos temas. Entonces eso hace falta en México también, no existe un grupo institucional de lactancia materna, se ha recomendado en reiteradas ocasiones justamente para aterrizar este y muchos otros temas relacionados con la promoción y la protección de la lactancia materna…” (Male, Civil Society Organization, [04C])* | *"...Something I want to share with you...is that there is an absence of public policy in the Mexican government that articulates breastfeeding efforts. So, the reality is that neither in the health sector nor in other sectors is there an articulation that allows us all to have clarity on how we join together with other, other allies.... So we have a gap and an absence of public policy that would help us coordinate these articulation processes..." (Male, Government, [06G])*  *"...We are missing a group of experts convened by a government institution, which in principle exists but they never convene. It was launched about two years ago and they have not met. So, in which different representatives from different sectors of the government, civil society, academia participate, so that these issues can be discussed. So this is also lacking in Mexico, there is no institutional group on breastfeeding, it has been recommended on several occasions just to implement this and many other issues related to the promotion and protection of breastfeeding..." (Male, Civil Society Organization, [04C]).* |
| Maternity cash transfer seen as helpful, but not sufficient | *“... No se estaría atacando el problema principal, o se sería como estar, cómo te diré. Que no estén en el sector formal, les está transgrediendo otros derechos. Entonces si por ejemplo, ese programa fuera de la Secretaría, si nosotros les diéramos ese dinero a las mujeres trabajadoras en el sector informal, pues sería como decir continúa en el sector informal está bien, te vamos a dar seis meses este dinero para que subsistas, para que puedas alimentarte y todo a tu bebé, pero después de los seis meses continúa en el sector informal. Entonces pues sería como, ni siquiera sería como, la solución para el problema. El problema en realidad no es no es tanto la… ¿Yo sé que el tema de del de la investigación es esto, no el de la lactancia materna, pero el problema principal que yo veo aquí es que están en el sector informal, no? Entonces darles dinero durante ese periodo pues sí les va a ayudar. Sí, va a ser una ayuda y todo, pero no va a acabar con su problema principal. ¿Qué va a pasar si ese bebé necesita una atención médica y ella no tiene derecho al IMSS? ¿Qué va a pasar si ella necesita atención médica? Si la guardería gratuita, no va a tener acceso a esa guardería gratuita por más que le paguen los seis meses de lactancia materna, entonces creo que sería no atacar el problema principal…”* *(Female, Government, [03G])*  *“...Sin duda, las mujeres que no tienen acceso al trabajo formal siguen requiriendo ese apoyo financiero, ese apoyo monetario, pero también el puro apoyo monetario no va a ser un factor que por sí mismo promueva, proteja y apoye a la lactancia materna de forma directa…” (Male, Government, [06G])*  *“...Por ejemplo, en zonas rurales, visitas domiciliarias por lo menos la primera semana posparto, aumenta la lactancia materna. Entonces sí va esta transferencia acompañada de esas consejerías y de esas intervenciones de lactancia que han sido exitosas, pues tiene mucho más potencial que solo a lo mejor darle la la transferencia. Pueden decir bueno, bueno yo hago con el dinero lo que quiera. No se les puede obligar obviamente a amamantar, pero si se les da la consejería se ha visto que las mujeres quieren amamantar [...] También que tengan acceso a salas de lactancia, dado que no tienen un lugar de trabajo en donde tengan salas de lactancia. [...] Tendría que ser como un paquete integral para estas mujeres que normalmente no tienen acceso en sus lugares de trabajo a este tipo de beneficios…” (Female, Academia, [02A]”*  *“...Lo que nos ha enseñado [el gobierno actual] es que si cambias de transferencia condicionada a transferencia directa, los objetivos de política pública no necesariamente se cumplen, porque estás hablando también de poblaciones que tienen muchos “constraints” en su vida. Entonces, si tienes lana adicional y que no la ligas absolutamente a nada, no necesariamente la vas a usar para mejorar la alimentación de tus hijas e hijos. No necesariamente la vas a usar para mejorar sus cuidados, lo vas a usar, literal la pirámide de Maslow, o sea para cumplir con tus necesidades básicas mucho más apremiantes. Entonces yo condicionaría también la transferencia…”* (*Female, Academia, [01A])*  *“...Yo creo que los apoyos económicos, como es una garantía de derechos, no se pueden condicionar, pero sí se pueden crear condiciones estructurales que orillen y que garanticen. Sigue siendo obligación del Estado garantizar que esos recursos se utilicen como se deberían. ¿Entonces, yo no puedo dar una transferencia económica a una mujer en posparto que no recibió educación en lactancia, que cree que amamantar en público está penado, que tuvo violencia obstétrica y salió con su bebé con un biberón del hospital y luego decirle ay, no lo utilizaste para la lactancia materna, por eso luego quitamos los programas, no? Porque seguimos diciendo las mujeres abandonan la lactancia. A ver, no, nosotros como sociedad estamos fallando en crear condiciones estructurales para que las mujeres que deciden amamantar logren hacerlo. Entonces, más allá del condicionamiento, yo le apostaría al flujo o a la ruta este que va a permear esa entrega para que las mujeres puedan además y o con tener acceso a la información como pasos decir tienes la información de esto, tienes esto, el otro y tu siguiente es este paso. Pero no, no narrado como una condición…”* *(Female, Civil Society Organization, [03C])*  *“...O sea, justo ellas no tienen tiempo de lactar porque necesitan ir a trabajar. Entonces creo que una transferencia monetaria ayuda mucho a que ellas ya no tengan esta incertidumbre, sino que estos meses ellas puedan tener el recurso para poderse enfocar a temas de lactancia y obviamente puede ser por más tiempo porque pues la idea es que sea un año. Pero pues a lo mejor si esa transferencia monetaria cubre algunos meses 1, 2, 3 o 6...”* *(Female, Government, [04G])*  *“... ¿Pero también pienso que este quizá una iniciativa así, aislada, aunque puede tener resultados positivos, pues debería entretejerse con otras iniciativas como por ejemplo la repartición del cuidado y la responsabilidad también de los padres. Sino de otra forma, aunque pues sí, sí se requiere que las mujeres tengan ese tiempo y ese espacio, pues para la lactancia y para su autocuidado. Pues si lo pensáramos solamente de esa forma, pues sí cae el riesgo de estar incentivando una política que refuerza la responsabilidad de las mujeres en el tema del cuidado y la lactancia…”* *(Female, Civil Society Organizations, [05C])*  *“...¿En ese sentido, me parece que entra esto que te estoy diciendo que es más allá de un programa social, que además son utilizados los programas sociales, muchas veces de manera clientelar, eh? Se han utilizado en las elecciones para manipular el voto de una manera o de otra. Debería de ser un derecho completamente institucionalizado si no hay de otra más que ese programa social, pues te diría que es bienvenido, porque siempre algún recurso va a ser bienvenido. Pero también te diría que ese recurso no soluciona la situación porque no es nada más, por ejemplo, en el caso de las trabajadoras de limpia, el tener un recurso de subsistencia es no perder su trabajo y esa vulnerabilidad no se la va a quitar el programa social. O sea, no se puede separar completamente una cosa de la otra. O sea, aquí es dignificar a las personas trabajadoras en el empleo informal en su totalidad. Una manera de dignificarla es sin duda, sobre todo a las mujeres, es proteger el todo lo relacionado con la maternidad, no porque son quienes se se pueden reproducir…” (Female, Civil Society Organization, [06C])*  *“...Bueno, desde mi perspectiva creo que no, quizá ese mecanismo quizá no sea, quizá no sea el adecuado. No, porque al final del día cada mujer decide si primero, si da lactancia y cuánto tiempo. Y tu no la puedes condicionar con determinado recurso, decirle no, pues este te vamos a pagar tanto y pero si tu das lactancia un año te vamos a dar una cuota y si es año y media otra cuota. No, porque entonces si no estás rompiendo ese derecho de ella, decidir hasta cuándo…”* *(Female, Government, [05G])* | *"...It would not be attacking the main problem, or it would be like being, how shall I put it? The fact that they are not in the formal sector is violating other rights. So, for example, if this program were part of the Ministry, if we were to give this money to working women in the informal sector, it would be like saying, continue in the informal sector, it is okay, we are going to give you this money for six months so that you can subsist, so that you can feed yourself and your baby, but after six months you continue in the informal sector. So it would be like, it wouldn't even be, like, the solution to the problem. The problem is actually not so much the… I know that the subject of the research is this, not breastfeeding, but the main problem that I see here is that they are in the informal sector, right? So, yes, giving them money during that period is going to help them. Yes, it will help them and everything, but it will not solve their main problem. What is going to happen if that baby needs medical attention and she is not entitled to IMSS? What is going to happen if she needs medical attention? If free daycare, she's not going to have access to that free daycare no matter how much they pay for her six months of breastfeeding, then I think that would be not attacking the main problem...." (Female, Government, [03G])*  *"...Without a doubt, women who do not have access to formal work still require that financial support, that monetary support, but also pure monetary support is not going to be a factor that by itself promotes, protects and supports breastfeeding in a direct way..." (Male, Government, [06G])*  *"...For example, in rural areas, home visits at least the first week postpartum increase breastfeeding. So if this transfer is accompanied by these counseling and breastfeeding interventions that have been successful, well, it has much more potential than just giving the transfer. They can say, well, I can do what I want with the money. Obviously you can't force them to breastfeed, but if you give them counseling, it has been seen that women want to breastfeed [...] Also that they have access to lactation rooms, since they don't have a workplace where they have lactation rooms. [...] It would have to be like a comprehensive package for these women who normally do not have access to this type of benefits in their workplaces..." (Female, Academia, [02A]"*  *"...What [the current government] has taught us is that if you change from conditional transfer to direct transfer, the public policy objectives are not necessarily met, because you are also talking about populations that have many constraints in their lives. So, if you have additional cash and you do not tie it to anything, you are not necessarily going to use it to improve the nutrition of your children. You are not necessarily going to use it to improve their care, you are going to use it, literally Maslow's pyramid, that is to say, to fulfill your most pressing basic needs. So I would condition the transfer as well..." (Female, Academy, [01A])*  *"...I believe that economic support, being a guarantee of rights, cannot be conditioned, but structural conditions can be created to guide and ensure that these resources are used as they should be. It is still an obligation of the State to guarantee that these resources are used properly. So, I cannot give an economic transfer to a postpartum woman who did not receive breastfeeding education, who believes that breastfeeding in public is punishable, who had obstetric violence and left the hospital with her baby with a bottle and then tell her oh, you did not use it for breastfeeding, that is why we then remove the programs, right? Because we keep saying women abandon breastfeeding. Let's see, no, we as a society are failing to create structural conditions so that women who decide to breastfeed are able to do so. So, beyond the conditioning, I would bet on the flow or the route that is going to allow women to have access to information as steps to say you have the information about this, you have this, the other and your next step is this. But no, not described as a condition..." (Female, Civil Society Organization, [03C])*  *“...I mean, they just don't have time to breastfeed because they need to go to work. So I think that a monetary transfer helps a lot so that they no longer have this uncertainty, but that instead these months they can have the resources to be able to focus on breastfeeding issues and obviously it can be for a longer period because the idea is that it should be a year. But maybe if this monetary transfer covers a few months 1, 2, 3 or 6..." (Female, Government, [04G]).*  *"...But I also think that perhaps such an isolated initiative, although it can have positive results, should be interwoven with other initiatives such as, for example, sharing caregiving responsibilities and the fathers’ responsibility. But in another way, although, yes, it is necessary for women to have that time and space for breastfeeding and for their self-care. Well, if we were to think of it only in that way, there is a risk that we would be encouraging a policy that reinforces women's responsibility in the area of caregiving and breastfeeding..." (Female, Civil Society Organizations, [05C])*  *"...In that sense, it seems to me that what I am telling you is more than a social program, that social programs are also used, many times to win people over, eh? They have been used in elections to manipulate the vote in one way or another. It should be a completely institutionalized right, if there is nothing else but that social program, then I would say that it is welcome, because some resource is always going to be welcome. But I would also say that this resource does not solve the situation because it is not just, for example, in the case of the cleaning workers, having a subsistence resource means not losing their job and this vulnerability is not going to be taken away by the social program. In other words, one thing cannot be completely separated from the other. In other words, here it is a matter of dignifying the workers in informal employment as a whole. One way to dignify them is undoubtedly, especially women, it is to protect everything related to maternity, not because they are the ones who can reproduce..." (Female, Civil Society Organization, [06C]).*  *" …Well, from my perspective I don't think so, maybe this mechanism is not, maybe it is not the right one. No, because at the end of the day, each woman decides first, if she breastfeeds, and for how long. And you cannot condition her with a certain resource, tell her, no, we are going to pay you so much, but if you breastfeed for a year we are going to give you one part and if it is a year and a half, we will give you another part. No, because then if you are not breaking her right to decide until when..." (Female, Government, [05G])* |

^1^ Quotations have been translated from Spanish to English as expressed by the participants.
