# Supplementary material for: Breastfeeding among women employed in Mexico’s informal sector: strategies to overcome key barriers
Source: Int J Equity Health. 2024 Jul 23;23:144. doi: 10.1186/s12939-024-02147-x (PMC11264414; doi:10.1186/s12939-024-02147-x)
Supplement: Supplementary file 5 — Supplementary Material 5 [file 12939_2024_2147_MOESM5_ESM.docx]

**Appendix Table S1.** Policy/program names, translations, and shorthands

| **Program/policy name in original Spanish** | **English translation** | **Acronym or shorthand** |
| --- | --- | --- |
| Atención Alimentaria en los Primeros 1000 Días | Nutrition Care in the First 1000 Days | Nutrition Care |
| Centros de Atención Infantil (CAI) | Childcare Centers | Public daycare program |
| Cursos en Línea Masivos del IMSS (CLIMSS) | IMSS (Mexican Social Security Fund) Online Courses | CLIMSS |
| [Día mundial de la donación de leche materna](https://www.gob.mx/salud/articulos/dia-mundial-de-la-donacion-de-leche-materna#:~:text=Se%20conmemora%20cada%2019%20de,participan%20en%20esta%20noble%20pr%C3%A1ctica.&text=Este%20alimento%20contiene%20la%20cantidad,de%20cualquier%20tipo%20de%20infecciones.) | World Human Milk Donation Day | WHMDD |
| Distintivo de Responsabilidad Laboral | Employment Responsibility Distinction | Employment Responsibility Distinction |
| Entornos Laborales Seguros y Saludables (ELSSA) | Healthy and Safe Workplace Environment | ELSSA |
| Estrategia Nacional de Atención a la Primera Infancia (ENAPI) | Strategy for Integrated Early Childhood Development | ENAPI |
| Hospital Amigo del Niño y de la Niña | Baby-friendly hospital | BFH |
| Instituto de Seguridad y Servicios Sociales de los Trabajadores del Estado (ISSSTE) | Institute for Social Security and Services for State Workers | ISSSTE |
| Instituto Mexicano de Seguro Social (IMSS) | Mexican Social Security Institute | IMSS |
| Programa de Salud Sexual y Reproductiva 2020-2024 | Sexual and Reproductive Health Program 2020-2024 | Sexual and Reproductive Health Program |
| Programa para el Bienestar de las Niñas y Niños, Hijos de Madres Trabajadoras | Wellbeing Program for Children of Working Mothers | Cash transfer program |
| Semana Mundial de Lactancia Materna | World Breastfeeding Week | WBW |
| Sistema Nacional de Cuidados | National Care System | National Care System |
